# Silyl‐Phosphino‐Carbene Complexes of Uranium(IV)

**DOI:** 10.1002/anie.201802080

**Published:** 2018-03-26

**Authors:** Erli Lu, Josef T. Boronski, Matthew Gregson, Ashley J. Wooles, Stephen T. Liddle

**Affiliations:** ^1^ School of Chemistry The University of Manchester Oxford Road Manchester M13 9PL UK

**Keywords:** alkylidenes, carbenes, metal–ligand multiple bonding, phosphines, uranium

## Abstract

Unprecedented silyl‐phosphino‐carbene complexes of uranium(IV) are presented, where before all covalent actinide–carbon double bonds were stabilised by phosphorus(V) substituents or restricted to matrix isolation experiments. Conversion of [U(BIPM^TMS^)(Cl)(μ‐Cl)_2_Li(THF)_2_] (**1**, BIPM^TMS^=C(PPh_2_NSiMe_3_)_2_) into [U(BIPM^TMS^)(Cl){CH(Ph)(SiMe_3_)}] (**2**), and addition of [Li{CH(SiMe_3_)(PPh_2_)}(THF)]/Me_2_NCH_2_CH_2_NMe_2_ (TMEDA) gave [U{C(SiMe_3_)(PPh_2_)}(BIPM^TMS^)(μ‐Cl)Li(TMEDA)(μ‐TMEDA)_0.5_]_2_ (**3**) by α‐hydrogen abstraction. Addition of 2,2,2‐cryptand or two equivalents of 4‐N,N‐dimethylaminopyridine (DMAP) to **3** gave [U{C(SiMe_3_)(PPh_2_)}(BIPM^TMS^)(Cl)][Li(2,2,2‐cryptand)] (**4**) or [U{C(SiMe_3_)(PPh_2_)}(BIPM^TMS^)(DMAP)_2_] (**5**). The characterisation data for **3**–**5** suggest that whilst there is evidence for 3‐centre P−C−U π‐bonding character, the U=C double bond component is dominant in each case. These U=C bonds are the closest to a true uranium alkylidene yet outside of matrix isolation experiments.

In contrast to the well‐developed nature of transition‐metal carbenes with covalent M=C double bonds, the analogous uranium chemistry is far more sparse.^1^ The first uranium carbene with a covalent U=C double bond, stabilised by one phosphorus(V) substituent, [U(CHPMe_2_Ph)(η^5^‐C_5_H_5_)_3_] (**I**),^2^ was reported in 1981 and its reactivity was well‐elaborated.^3^ After a pause of some three decades the area was revived with various examples of uranium–carbene complexes with one or two phosphorus(V) substituents that stabilise the carbene.^4^ The majority of these complexes exhibit covalent U=C double‐bond interactions, that is, uranium plays a significant role in stabilising the carbene by accepting charge from it, but in all cases the phosphorus(V) substituents introduce the competing carbene and ylide resonance forms R_3_P^+^−C(R)=U^−^ ↔ R_3_P=C(R)−U (R=H or R′_3_P), where in the latter the phosphorus(V) substituent plays a significant stabilising role by accepting charge from the carbene. So, those U=C double bonds are not as fully developed as they might otherwise be.1a,1e

Apart from fleeting reactive intermediates,^5^ the only reports of unfettered uranium–carbon multiple bonds pertain to fundamental species such as [U≡C], [C≡U≡C], [U≡CH], [C≣U=O], [F_3_U≡CH], and [X_2_U=CH_2_] (X=H, F, Cl),^6^ prepared on microscopic scales in matrix isolation experiments at cryogenic temperatures (<10 K). Thus, the synthesis of a covalent U=C double bond, where the carbene substituents do not significantly affect the U=C component, in a true uranium alkylidene is yet to be reported under ambient conditions after synthetic efforts spanning four decades.1a,1e, 2 Without exception, outside of matrix isolation all uranium carbenes with covalent U=C double bonds are stabilised with phosphorus(V) substituents,1a,1e which has posed the question as to whether U=C double bonds free of phosphorus(V) substituents are accessible under ambient conditions. A full understanding of U=C double bonds is thus lacking, but is key to informing the ongoing debate over the nature of actinide chemical bonding and to providing organouranium reactivity benchmarks.

The complex [Sc{C(SiMe_3_)(PPh_2_)}{HC(MeCNAr)_2_}(THF)] (**II**, Ar=2,6‐diisopropylphenyl) was recently reported.^7^ In this compound, the Sc=C bond is highly polarised, and consequently a π‐delocalised Sc−C−P 3‐centre unit is found. Inspired by that report, and related early d‐block analogues,^8^ we reasoned that using {C(SiMe_3_)(PPh_2_)}^2−^, never before deployed in actinide chemistry, might present, if synthetically accessible, a U=C double bond that would be more fully developed than in phosphorus(V)‐substituted variants because the phosphorus(III) substituent should be less able to accept charge from the carbene. This U=C double bond might thus be anticipated to be closer to matrix isolation examples,^6^ since 5f uranium(IV) might be expected to better stabilise the carbene than 3d scandium(III).

We report herein the synthesis, characterisation, and reactivity benchmarking of silyl‐phosphino‐carbene complexes of uranium(IV). Outside of matrix isolation these are the first examples of covalent actinide–carbon double bonds prepared without phosphorus(V) substituents. Our strategy exploited α‐hydrogen abstraction, and so they are a significant advance towards isolating a true uranium alkylidene under ambient conditions. In contrast to **II**,^7^ whilst we find evidence for 3‐centre P−C−U π‐bonding character, the U=C double bond component is dominant because the uranium ions are the dominant acceptor of charge from the carbene. So, these U=C bonds can be considered to be the closest to a true uranium alkylidene thus far prepared outside of matrix isolation experiments.

After extensive screening of multiple types and combinations of alkyl ligands (for example, CH_3_, CH_2_Bu^t^, CH_2_SiMe_3_, CH(SiMe_3_)_2_, CH_2_C_6_H_5_, CH(C_6_H_5_)_2_, none of which facilitate α‐hydrogen abstraction in any combinations nor under thermolysis or photolysis conditions) we deduced^9^ that installation of {PhC(H)SiMe_3_}^−^ at uranium in [U(BIPM^TMS^)(Cl)(μ‐Cl)_2_Li(THF)_2_] (**1**, BIPM^TMS^=C(PPh_2_NSiMe_3_)_2_)4j produces the carbene precursor complex [U(BIPM^TMS^)(Cl){CH(Ph)(SiMe_3_)}] (**2**; Scheme 1). Complex **2** is best used in situ, and when treated with [Li{CH(SiMe_3_)(PPh_2_)}(THF)]^10^ with *N*,*N*,*N*′,*N*′‐tetramethylethylenediamine (TMEDA) elimination of PhCH_2_SiMe_3_ by α‐hydrogen abstraction results in isolation of the red complex [U{C(SiMe_3_)(PPh_2_)}(BIPM^TMS^)(μ‐Cl)Li(TMEDA)(μ‐TMEDA)_0.5_]_2_ (**3**) in 36 % crystalline yield (Scheme 1). It would seem that the occluded (TMEDA)_1.5_LiCl fragment acts as a protecting group blocking the coordination site left otherwise vacant by the eliminated PhCH_2_SiMe_3_, preventing decomposition or dimerisation.

**Scheme 1:**
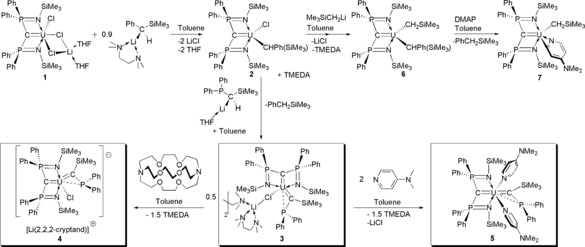
Synthesis of the uranium(IV)–carbene complexes **3**, **4**, and **5** from precursors **1** and **2**, and sequential alkylation of **2** (to give **6**) and reactivity of **6** with 4‐*N*,*N*‐dimethylaminopyridine (DMAP) to give the C−H activated product **7**,which contrasts with the adduct formation of **5**.

Addition of 2,2,2‐cryptand to **3** eliminates the TMEDA to give [U{C(SiMe_3_)(PPh_2_)}(BIPM^TMS^)(Cl)][Li(2,2,2‐cryptand)] (**4**). Alternatively, treatment of **3** with two equivalents of 4‐*N*,*N*‐dimethylaminopyridine (DMAP) eliminates the (TMEDA)_1.5_LiCl entirely to yield [U{C(SiMe_3_)(PPh_2_)}(BIPM^TMS^)(DMAP)_2_] (**5**). Complexes **4** and **5** are isolated as red crystalline solids in 86 and 65 % yields, respectively (Scheme 1).^9^

The solid‐state molecular structures of **3**–**5** were determined,^9^ and **5** is shown in Figure 1. The salient features of **3**–**5** are the presence of a meridionally coordinated BIPM^TMS^ ligand and a silyl‐phosphino‐carbene ligand to uranium.

**Figure 1:**
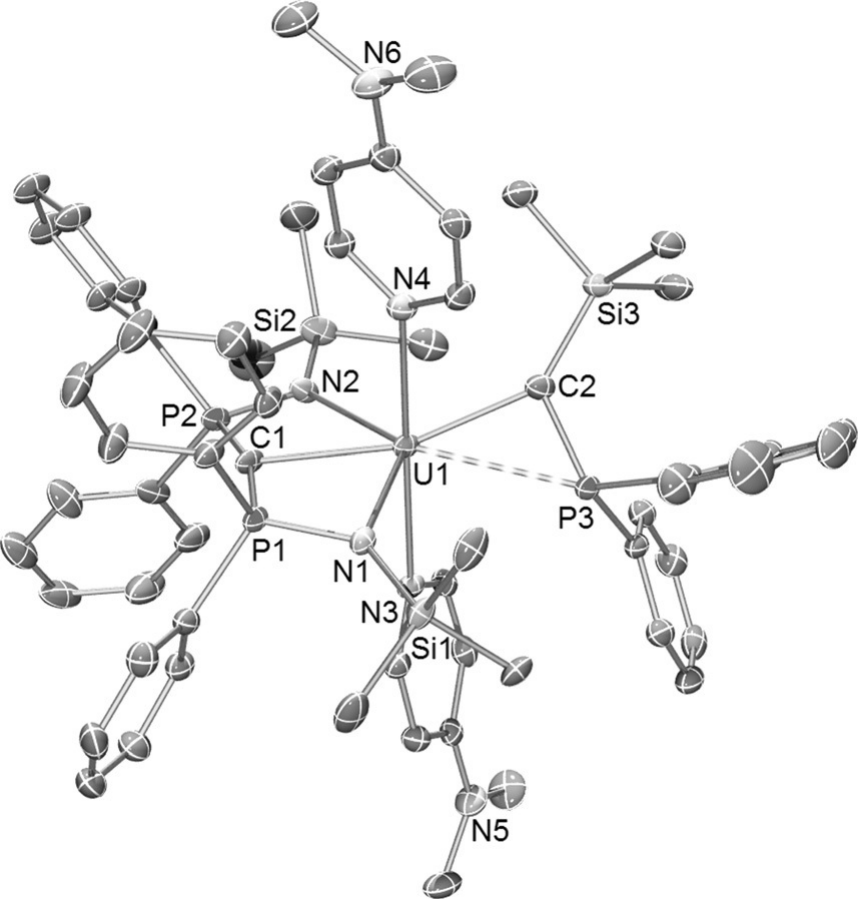
Molecular structure of **5** at 150 K with ellipsoids set at 40 % probability. Hydrogen atoms, minor disorder components, and lattice solvent are omitted for clarity. The weak U=C−P interaction is represented by a dashed bond between uranium and phosphorus.^22^

In **3**–**5**, the U=C_carbene_/U=C_BIPM_ distances are 2.270(10)/2.405(9), 2.265(2)/2.459(2), and 2.296(5)/2.424(5) Å, respectively. Considering the different uranium coordination environments and formal charge states, these U=C_carbene_ distances are invariant and short. In contrast, the longer but typical U=C_BIPM_ bond lengths vary more, suggesting that the U=C_carbene_ unit is the more robust, structure‐dictating unit. The U=C_carbene_ distances are in between the sum of covalent uranium–carbon single (2.45 Å) and double (2.01 Å) bond radii,^11^ and fit with the trend of uranium–carbon quadruple ([C≣U=O], 1.77 Å), triple ([F_3_U≡CH], 1.94 Å), and double ([F_2_U=CH_2_], 2.07 Å) bond distances found computationally^6^ when considering the major differences in these species of uranium coordination number, oxidation state, and steric encumbrance. Considering their differing natures, the U=C_carbene_ distances in **3**–**5** compare very well to the U=C distances in **I** (U^IV^, 2.293(2) Å),^2^ [U(CHPPh_3_){N(SiMe_3_)_2_}_3_] (U^IV^, 2.278(8) Å),4i **1** (U^IV^, 2.310(4) Å), [U(BIPM^TMS^)(Cl)_2_(I)] (U^V^, 2.268(10) Å), and [U(BIPM^TMS^)(O)(Cl)_2_] (U^VI^, 2.183(3) Å).4h,4j

The U⋅⋅⋅P distances in **3**–**5** are 2.774(3), 2.8277(5), and 2.8371(13) Å, respectively, and are at the limit of, or exceed, the covalent single bond radii of uranium and phosphorus (2.81 Å).^11^ Further, it is clear from the solid‐state structures that owing to the orientations of the Ph_2_P groups the phosphorus lone pairs do not point towards the uranium ions in **3**–**5**. However, there is clearly a U−P bond in [U(PH_2_){N(CH_2_CH_2_NSiPr^i^_3_)_3_}] even though the U−P distance in that complex is 2.883(2) Å,^12^ and the U=C−P angles in **3**–**5** are acute (ca. 88°), and the U=C−Si angles are obtuse (ca. 140°; Si−C−P angles ca. 132°). It is interesting to note that in sterically unencumbered alkylidenes such as [X_2_U=CH_2_] (X=H, F, Cl, Br, I)6e,6g,6h one of the U=C−H angles is also about 88°. On balance, we surmise that there are U⋅⋅⋅P interactions in **3**–**5**, but owing to the geometric disposition they must be weak. We note that the P−C_carbene_ distances are relative short (for example, 1.739(5) Å in **5**, cf 1.743(3) Å in **II** 7), which suggests some P−C negative hyperconjugation and thus some phosphorus π‐stabilisation of the carbene.

The ^1^H NMR spectra of **3**–**5** span the ranges −32 to +25, −33 to +59, and −16 to +48 ppm, respectively. The ^31^P NMR spectra of **3**–**5** reveal broad BIPM^TMS^ phosphorus resonances at −598, −582, and −402 ppm, respectively, but the phosphine resonances could not be located. Both sets of NMR data are characteristic of 5f^2^ uranium(IV)–BIPM^TMS^ complexes.4b,4c,4d Owing to low solubilities post‐crystallisation, reliable UV/Vis/NIR spectra of **3** and **4** could not be obtained. However, the spectrum of **5** 9 is consistent with the uranium(IV) formulation.1b, 4l, 13 The ATR‐IR spectra of **3**–**5** all exhibit strong absorptions at about 650 and about 595 cm^−1^, which are shown by analytical frequency calculations, computed to within 25 cm^−1^ of experiment in each case, to be the main U=C_carbene_ bond stretches in **3**–**5**.

Confirmation of the uranium(IV) assignments of **3**–**5** is provided by SQUID magnetometry.^9^ The magnetic moments per uranium ion of **3**–**5** are all about 3.0 μ_B_ at 298 K, in each case changing little until about 50 K where the magnetic moment drops sharply to about 0.8 μ_B_ by 2 K and is tending to zero. The magnetic moment of uranium(IV) usually smoothly decreases over the temperature range 298 to 2 K and tends to zero as this ion is a magnetic singlet at low temperature with a residual magnetic moment from temperature‐independent paramagnetism (ca. 0.4 μ_B_).1b, 13, 14 The retention of higher than usual magnetic moments until 50 K and also at 2 K is atypical of most uranium(IV) magnetism, but is characteristic of cases where one or more strongly donating multiply bonded ligands are coordinated to uranium(IV).4a–4c, 15, 16

To probe the U=C_carbene_ linkages in **3**–**5**, we modelled them with DFT.^9^ We replaced the bridging TMEDA in **3** with a NMe_3_ surrogate to provide the computationally tractable monomer model [U{C(SiMe_3_)(PPh_2_)}(BIPM^TMS^)(μ‐Cl)Li(TMEDA)(NMe_3_)] (**3′**) whilst retaining the charge balance and steric profile, we computed the full [U{C(SiMe_3_)(PPh_2_)}(BIPM^TMS^)(Cl)]^−^ anion component of **4** (**4^−^**), and used the full model of **5**. The geometry optimised structures of **3′**, **4^−^**, and **5** are in excellent agreement with their experimental structures (Table 1), and we include data for **I** for comparison.1a, 4f The computed U and C charges are consistent with their formulations.

**Table 1:** Selected computed DFT, NBO, and QTAIM data for the U=C bonds in **3′**, **4^−^**, **5**, and **I**.

	Bond length and index^[b,c]^		Charges		NBO σ‐component^[f]^			NBO π‐component^[f]^			QTAIM^[g]^			
Entry^[a]^	U=C	BI	*q*_U_^[d]^	*q*_C_^[e]^	U[%]	C[%]	U 7s/7p/6d/5f	U[%]	C[%]	U 7s/7p/6d/5f	*ρ*(*r*)	∇^2^ *ρ*(*r*)	*H*(*r*)	*ϵ(r*)
**3′**	2.277	1.78	2.87	−1.88	19	81	2:1:53:44	20	80	0:0:19:81	0.12	0.13	−0.04	0.52
	2.392	1.26		−2.00	14	86	1:0:32:67	11	89	0:0:33:67	0.08	0.06	−0.02	0.26
**4^−^**	2.286	1.71	2.69	−1.95	15	85	0:1:54:45	13	87	0:0:21:79	0.11	0.12	−0.04	0.48
	2.448	1.13		−1.79	11	89	0:0:38:62	8	92	1:1:31:67	0.08	0.08	−0.02	0.26
**5**	2.273	1.78	3.10	−2.02	19	81	0:0:42:58	21	79	0:0:35:65	0.12	0.11	−0.05	0.46
	2.394	1.25		−1.84	15	85	0:0:30:70	13	87	0:0:36:64	0.09	0.12	−0.03	0.22
**I**	2.354	1.64	2.49	−1.97	0	100	–	25	75	0:0:6:94	0.09	0.14	−0.03	0.25

[a] All molecules geometry optimised without symmetry constraints at the LDA VWN BP86 TZP/ZORA level; for **3′**, **4^−^**, and **5** the first entry is the U=C_carbene_ bond and the second entry is the U=C_BIPM_ bond. [b] Calculated U=C distances [Å]. [c] U=C Nalewajski–Mrozek bond indices. [d] MDC‐q charge on U. [e] MDC‐q charge on carbene carbon. [f] Natural bond orbital (NBO) analyses. [g] QTAIM topological electron density [*ρ*(*r*)], Laplacian [∇^2^ *ρ*(*r*)], electronic energy density [*H*(r)], and ellipticity [*ϵ*(*r*)] bond critical point data.

For **3′**, **4^−^**, and **5** the HOMO and HOMO−1 are singularly occupied and of essentially pure 5f character. The next orbitals in each case, which are doubly occupied, are the U=C_carbene_ π‐bond (HOMO−2), followed by the U=C_carbene_ σ‐bond (HOMO−3). Slightly lower in energy in the HOMO−4 to HOMO−8 regions are the U=C_BIPM_ π‐ then σ‐bonds. However, in all complexes there is extensive and variable mixing of orbital contributions from the U=C_BIPM_, U=C_carbene_, and phosphine lone pairs, so, since other orbital coefficients also intrude into these molecular orbitals, the overall bonding picture of these energetically similar orbitals is convoluted by the inherently delocalised nature of the DFT calculations.

To obtain a localised, more chemically intuitive description of the bonding in **3′**, **4^−^**, and **5** we turned to NBO analysis, Table 1. The U=C_carbene_ σ‐ and π‐bonds in **3′** and **5** are remarkably similar and for charge‐rich **4^−^** the σ‐ and π‐bonds show lower uranium contributions. We conclude that the 6d and 5f contributions to the U=C_carbene_ σ‐bonds are generally fairly equal, but for the corresponding π‐bonds 5f contributions dominate these more angular interactions. The data for **3′**, **4^−^**, and **5** are similar to computed data for simpler, fundamental [X_2_U=CH_2_] (X=F, Cl) species prepared in matrix‐isolation experiments,6a,6e,6g where average uranium σ‐ and π‐contributions to those U=C double bonds of about 21 and about 26 % are found. It is also instructive to compare **I** to the U=C_carbene_ units in **3′**, **4^−^**, and **5**; for **I** the σ‐bond is essentially electrostatic, but the π‐bond is slightly more covalent. The U=C_carbene_ bonds can also be internally compared to the U=C_BIPM_ cases within each of **3′**, **4^−^**, and **5**, and we note that the uranium contributions to the U=C_BIPM_ bonds are consistently 4–9 % lower than the corresponding U=C_carbene_ for each pair. We also note that the U=C_BIPM_ uranium contributions are lower than in other uranium(IV)–BIPM^TMS^ complexes,1a presumably reflecting the strongly donating nature of the silyl‐phosphino‐carbene.

Nalewajski–Mrozek bond order analyses (Table 1) reveals U=C_carbene_ bond orders that are consistently higher than the U=C_BIPM_ bond orders, which are slightly lower than usually found for uranium(IV)–BIPM^TMS^ complexes,1a underscoring the strongly donating nature of the carbene group. The U=C_carbene_ values are also higher than for **I** and bond orders of about 1.45 for [X_2_U=CH_2_] (X=F, Cl).6a,6d For comparison, the BIPM^TMS^ imino donors exhibit U−N bond orders of about 0.8, the coordinated DMAP ligands in **5** exhibit U−N bond orders of about 0.6, and the phosphine U−P bond orders vary from about 0.3 in **3** and **4** (which derives from indirect mixing of the phosphine orbitals into the uranium–carbene bonding orbitals rather than any direct U−P interaction) to <0.1 in **5**.^17^ Supporting this latter point, the P−C_carbene_ bond orders average 1.20, reflecting the aforementioned mixing by negative hyperconjugation. So, some 3‐centre U−C−P π‐topology is found in **3**–**5**, however the U=C double bonds in **3**–**5** with U=C bond orders about 1.5 times the P−C bond orders contrast to the more delocalised 3‐centre Sc−C−P π‐bonding scenario in **II** where the situation is reversed with the C−P bond order about 1.6 times the Sc=C bond order.^7^ Thus, the bonding situation in **3**–**5** is closer to the localised one found in [Ta(CHPMe_2_)(η^5^‐C_5_Me_5_)_2_(PMe_3_)]8f than in **II**.^7^ This underscores the key, dominant role of uranium stabilisation of the carbenes in **3**–**5** that is also rather different to the situation found in related free carbenes such as Me_3_SiCP(NPr^i^_2_)_2_.^18^

Along with orbital‐based DFT and NBO methods, we performed a topological bond analysis using QTAIM, Table 1.^19^ For a chemical bond at the bond critical point (BCP) the topological electron density (*ρ*(*r*)) tends to be <0.1 when the bond is ionic and >0.2 when it is covalent. For all complexes U=C BCPs were found with *ρ*(*r*) values ordered U=C_carbene_ > U=C_BIPM_≈**I**, indicating the presence of covalent uranium–carbon chemical bonds, albeit polarised ones. Single or triple bonds present cylindrical distributions of electron density around the inter‐nuclear bond axis at the BCP (*ϵ*(*r*)=0). Double bonds, however, are asymmetric when viewed down the inter‐nuclear bond axis (*ϵ*(*r*)>0). For comparison, the carbon–carbon bonds in ethane, benzene, and ethylene have *ϵ*(*r*) values of 0, 0.23, and 0.45, and transition metal–alkylidene complexes generally have *ϵ*(*r*) values of about 0.5.^20^ The QTAIM analysis consistently returns non‐zero U=C_carbene_ and U=C_BIPM_ ellipticities, thus both are clearly U=C double‐bond interactions but with the former clearly better developed than the latter, and this is in line with those of **I** and uranium–BIPM complexes generally.1a The P−C_carbene_ *ϵ*(*r*) values of **3**–**5** are consistently about 0.1, which only deviating modestly from zero gives clarity over the true extent of negative hyperconjugation and 3‐centre U−C−P π‐character that could be otherwise overestimated from visual inspection of molecular orbitals alone. Interestingly, no U−P BCPs are found in **3**–**5**. Since there are no U−P BCPs, and the structural and NBO data suggest phosphine lone pairs that point away, not to, uranium, it is concluded that any U⋅⋅⋅P interactions must be relatively weak. Furthermore, ring CPs between the BIPM^TMS^ phosphorus centres and uranium ions in **3′**, **4^−^**, and **5** are found by QTAIM, and we have found U−P BCPs in other compounds with U−P bonds,^12^, ^17^ suggesting that the absence of uranium–phosphine BCPs in three independent calculations is not spurious.

Experimentally, it is interesting to note that addition of DMAP to **3** only forms the DMAP adduct **5**, whereas addition of DMAP to **II** 7 results in rapid C−H activation of DMAP. The coordination of two DMAP molecules in **5** suggests that there are no steric barriers, and thus the lack of DMAP C−H activation by **3** experimentally supports the notion that the U=C_carbene_ bonds in **3**–**5** are more covalent, and thus less reactive units than that in **II**.^7^ In support of this notion, when **2** is converted into [U(BIPM^TMS^){CH(Ph)(SiMe_3_)}(CH_2_SiMe_3_)] (**6**), which does not undergo α‐hydrogen abstraction, and then treated with DMAP C−H activation occurs under mild conditions to give [U(BIPM^TMS^)(NC_5_H_3_‐4‐NMe_2_)(CH_2_SiMe_3_)] (**7**), Scheme 1.^9^ This underscores the more basic, ionic nature of U−C single bonds compared to U=C double bonds.

Preliminary reactivity studies reveal divergent carbene‐ and phosphine‐centred reactivities (Scheme 2). Complexes **3**–**5** all react with benzaldehyde and 9‐anthracenecarboxaldehyde to produce alkenes by Wittig‐type chemistry. Two equivalents of aldehyde are consumed per uranium each time, irrespective of reactant ratios, to produce (Ph_2_P)(Me_3_Si)C=C(H)(R′) and (Me_3_SiNPPh_2_)_2_C=C(H)(R′) (R′=anthracene or phenyl). Potentially of more interest, **3** reacts with PhCCPh to give [U{C(SiMe_3_)(Ph_2_PCPhCPh)}(BIPM^TMS^)] (**8**) where the alkyne has formed a metallocycle between the phosphine and uranium centres. This complex is notable on two counts. The U=C_carbene_ double bond is so robust that reactivity has preferentially occurred at the phosphine, and indeed the U=C_carbene_ distance of 2.316(7) Å in **8** is by the 3σ‐criterion barely perturbed from **3**–**5** whilst the U=C_BIPM_ distance (2.405(7) Å) is comparable to that in **3**. Despite the fact there is clearly a vacant coordination site *trans* to the alkenyl unit in **8** the carbene resides essentially *trans* to the central BIPM^TMS^ carbon (C=U=C 173.8(2)°) even though there is no obvious constraining steric reason for it to do so. If the *trans* influence is operating here this would not be expected since there is clearly space for the C=U=C angle to decrease further, and this hints at the possible presence of an inverse *trans* influence.4a–4c, 21

**Scheme 2:**
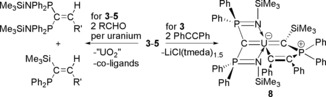
Synthesis of the Wittig alkene products and **8** from complexes **3**–**5**. R′=phenyl or 9‐anthracene.

To conclude, by utilising a silyl‐phosphino‐carbene we have prepared three uranium(IV) carbenes by α‐hydrogen abstraction. These are the first actinide–carbon double bonds outside of matrix isolation conditions to be free of phosphorus(V) substituents, and the first use of such a ligand in f‐block chemistry; as such they exhibit uranium(IV)–carbon bond distances that are amongst the shortest on record. Although there is evidence for the presence of a 3‐centre U−C−P π‐interaction facilitated by negative hyperconjugation, the characterisation data all suggest the presence of U=C_carbene_ double bonds that dominate the bonding picture. These U=C_carbene_ bonds can be considered to be the closest to a true uranium alkylidene yet prepared outside of matrix isolation experiments. Complexes **3**–**5** take us a step further towards isolable uranium alkylidenes, and preliminary reactivity studies have revealed divergent carbene‐ and phosphine‐centred reactivities.

## Conflict of interest

The authors declare no conflict of interest.

## Supporting information

As a service to our authors and readers, this journal provides supporting information supplied by the authors. Such materials are peer reviewed and may be re‐organized for online delivery, but are not copy‐edited or typeset. Technical support issues arising from supporting information (other than missing files) should be addressed to the authors.

Supplementary
