# Supplementary material for: Silyl‐Phosphino‐Carbene Complexes of Uranium(IV)
Source: Angew Chem Int Ed Engl. 2018 Mar 26;57(19):5506–11. doi: 10.1002/anie.201802080 (PMC6001699; doi:10.1002/anie.201802080)
Supplement: Supplementary file 1 — Supplementary [file ANIE-57-5506-s001.pdf]

Angewandte Chemie
Eine Zeitschrift der Gesellschaft Deutscher Chemiker

Supporting Information

Silyl-Phosphino-Carbene Complexes of Uranium(IV)

*Erli Lu, Josef T. Boronski, Matthew Gregson, Ashley J. Wooles, and Stephen T. Liddle**

anie_201802080_sm_miscellaneous_information.pdf

Experimental

General

All manipulations were carried out using Schlenk techniques, or an MBraun UniLab glovebox, under an atmosphere of dry nitrogen. Solvents were dried by passage through activated alumina towers and degassed before use. All solvents were stored over potassium mirrors except for THF which was stored over activated 4 Å sieves. D-solvent was distilled from potassium, degassed by three freeze-pump-thaw cycles and stored under nitrogen. [U(BIPM^{TMS})(Cl)(μ-Cl)$_2$Li(THF)$_2$] (**1**),[1] [Li{CH(Ph)(SiMe$_3$)}(TMEDA)],[2] and [Li{CH(SiMe$_3$)(PPh$_2$)}(THF)][3] were synthesized as previously reported. 4,7,13,16,21,24-Hexaoxa-1,10-diazabicyclo[8.8.8]hexacosane (2,2,2-cryptand), *N,N*-dimethylaminopyridine (DMAP), benzaldehyde, and 9-anthracenecarboxaldehyde were purchased from Sigma Aldrich, dissolved in dry diethyl ether, dried over activated 4 Å molecular sieves, then decanted and the diethyl ether removed to use.

^{1}H, ^{7}Li, ^{13}C, ^{29}Si, and ^{31}P NMR spectra were recorded on a Bruker 400 spectrometer operating at 400.2, 155.5, 100.6, 79.5, and 162.0 MHz respectively; chemical shifts are quoted in ppm and are relative to tetramethylsilane (^{1}H, ^{13}C, ^{29}Si), external 85% H_3PO_4 (^{31}P), and 1M LiCl (^{7}Li). Attenuated total reflectance infrared spectra were recorded on a Bruker Alpha spectrometer with Platinum-ATR module. UV/Vis/NIR spectra were recorded on a Perkin Elmer Lambda 750 spectrometer. Data were collected in 1mm path length cuvettes loaded in an MBraun UniLab glovebox and were run versus the appropriate toluene reference solvent. Variable-temperature magnetic moment data were recorded in an applied dc field of 0.1 or 0.5 T on a Quantum Design MPMS XL7 superconducting quantum interference device magnetometer using powdered samples. Care was taken to ensure compound purity and complete thermalisation of the sample before each data point was measured and samples were immobilised in an eicosane matrix to prevent sample reorientation during measurements. Elemental microanalyses were carried out by Mr Martin Jennings at the Micro Analytical Laboratory, School of Chemistry, The University of Manchester.

Preparation of [U{C(SiMe$_3$)(PPh$_2$)}(BIPM^{TMS})(μ-Cl)Li(TMEDA)(μ-TMEDA)$_{0.5}$]$_2$ (3)

Under N_2 atmosphere at −78 °C, [Li{CH(Ph)(SiMe$_3$)}(TMEDA)] (1.29 g, 4.50 mmol) in toluene (20 ml) was very slowly introduced into a stirring suspension of **1** (5.44 g, 5.00 mmol) in toluene (20 ml), in a dropwise manner. The addition was completed after 2 hours. The mixture was stirred at −78 °C for a further 30 minutes, and then stirred at room temperature for 1 hour. The dark red solution was then filtered, and the red filtrate, which now contains **2** as the major product, was used in the next step. The dark red filtrate from the previous step was cooled to −78 °C and [Li{CH(SiMe$_3$)(PPh$_2$)}(THF)] (1.40 g, 4.00 mmol) in toluene (20 ml) was added dropwise. The dark red mixture was stirred at −78 °C for 15 minutes, and then stirred at room temperature for 24 hours. The mixture was then filtered and the resulting dark red filtrate was concentrated to approximately 4 ml and then stored at –35 °C for 1 day, affording **3** as red crystals. Yield: 2.43 g, 36%. Single crystals suitable for X-ray diffraction were grown form concentrated toluene solution at −35 °C. Once obtained as crystals, **3** is not soluble in aromatic and aliphatic hydrocarbon solvents, and decomposes in coordinative and polar solvents. Thus, the NMR spectra of **3** were recorded from NMR scale reaction between **2** and 1 equivalent of [Li{CH(SiMe$_3$)(PPh$_2$)}(THF)] in C_6D_6, along with the formation of PhCH$_2$SiMe$_3$. However, this meant that sufficiently concentrated solutions for ^{13}C and ^{29}Si NMR spectra could not be prepared. A reliable electronic absorption spectrum is not available due to the same reason. Anal. Calcd for $C_{112}H_{162}Cl_2Li_2N_{10}P_6Si_6U_2 \cdot C_7H_8$: C 53.82; H 6.45; N 5.27%. Found: C 53.20; H 6.39; N 5.00%. ^{1}H NMR (C_6D_6, 298 K): δ −31.59 (s, 18 H, SiC*H*$_3$), −17.89 (s, 4.5 H), −5.10 (br, 15.5 H), −4.00 (br, 9 H), −2.85 (s, 5 H), −1.55 (s, 8 H), −1.00 (s, 2 H), −0.29 (br, 7 H), −0.06 (s, 9 H, SiC*H*$_3$ of PhCH$_2$SiMe$_3$), 1.94 (s, 2 H, C*H*$_2$ of PhCH$_2$SiMe$_3$), 4.86 (s, 2 H), 5.42 (s, 3 H), 20.88 (s, 3 H), 24.60 (s, 4 H). ^{31}P NMR (C_6D_6, 298 K): δ −598.23 (br, U=C(*P*NSiMe$_3$Ph$_2$)$_2$). ATR-IR ν/cm^{-1}: 417 (m), 450 (s), 466 (w), 489 (m), 508 (s), 523 (s), 593 (s), 616 (w), 630 (s), 650 (s), 693 (s), 710 (s), 725 (s), 735 (w), 828 (s), 948 (w), 1014 (m),

1027 (m), 1067 (s), 1091 (s), 1159 (w), 1230 (m), 1243 (s), 1282 (w), 1305 (w), 1339 (s), 1433 (s), 1469 (m), 2840 (w), 2881 (w), 2943 (w), 3049 (w).

Preparation of [U{C(SiMe$_3$)(PPh$_2$) (BIPM^{TMS})(Cl)][Li(2,2,2-cryptand)] (4)

Under N_2 atmosphere at room temperature, 2,2,2-cryptand (0.14 g, 0.38 mmol) in toluene (2 ml) was added into a suspension of **3** (0.50 g, 0.19 mmol) in toluene (3 ml). A dark red solution was formed immediately after mixing, and then red crystalline solid precipitated out after standing at room temperature for 5 minutes. The yellow mother liquor was removed and the red crystalline solid was washed with pentane (3 × 2 ml) and dried *in vacuo*, to afford **4** as a red crystalline solid. Yield: 0.48 g, 86%. Single crystals suitable for X-ray diffraction were grown from C_6D_6 solution at room temperature. Once obtained as crystals, **4** is not soluble in aromatic and aliphatic hydrocarbon solvents, and decomposes in coordinative and polar solvents. Thus, the NMR spectra of **4** were recorded from NMR scale reaction between **3** and 2 equivalents of 2,2,2-cryptand. However, this meant that sufficiently concentrated solutions for ^{13}C and ^{29}Si NMR spectra could not be prepared. A reliable electronic absorption spectrum is not available due to the same reason. Anal. Calcd for $C_{47}H_{57}ClN_2P_3Si_3U.C_{18}H_{36}LiN_2O_6$: C 52.61; H 6.32; N 3.78%. Found: C 52.75; H 6.44; N 3.89%. ^{1}H NMR (C_6D_6, 298 K): δ −32.51 (s, 18 H, SiC*H$_3$*), −17.39 (s, 4 H), −2.80 (s, 4 H), −2.01 (s, 12 H), −1.68 (s, 13 H), −1.29 (s, 12 H), −1.04 (t, 1 H, $^3J_{HH}$ = 6.4 Hz, Ar*H*), 2.13 (s, 15 H), 2.37 (s, 4 H), 2.55 (t, 2 H, $^3J_{PH}$ = 6.5 Hz, H_{ortho} of P-*Ph*), 3.52 (t, 2 H, $^3J_{HH}$ = 5.4 Hz, C*H$_2$* of 2, 2, 2-cryptand), 3.54 (br, 1H) 3.67 (s, 2 H), 3.80 (br, 4 H), 3.93 (t, 2 H, $^3J_{HH}$ = 5.5 Hz, C*H$_2$* of 2, 2, 2-cryptand), 4.12 (br, 4 H), 20.35 (t, 2 H, $^3J_{HH}$ = 7.0 Hz, Ar*H*), 24.19 (d, 4 H, $^3J_{HH}$ = 6.8 Hz, Ar*H*), 45.28 (s, 9 H, SiC*H$_3$*), 58.66 (s, 4 H). ^{31}P NMR (C_6D_6, 298 K): δ −581.69 (br, U=C(*P*NSiMe$_3$Ph$_2$)$_2$). ^{7}Li NMR (C_6D_6, 298 K): δ –6.54 (s). ATR-IR v/cm^{-1}: 419 (w), 441 (m), 469 (m), 499 (m), 519 (s), 598 (s), 617 (w), 630 (m), 652 (m), 695 (s), 726 (w), 741 (s), 759 (s), 826 (s), 934 (w), 1037 (m), 1064 (s), 1088 (s), 1106 (s), 1144 (w), 1241 (s), 1304 (w), 1341 (s), 1378 (w), 1432 (m), 1473 (w), 1578 (w), 2881 (w), 2929 (w), 3052 (w).

Preparation of [U{C(SiMe$_3$)(PPh$_2$)}(BIPM^{TMS})(DMAP)$_2$] (5)

Under N_2 atmosphere at room temperature, 4,4-dimethylaminopyridine (0.06 g, 0.45 mmol) in toluene (3 ml) was added into a suspension of **3** (0.30 g, 0.11 mmol) in toluene (3 m). After stirring at room temperature for 1 hour, the mixture was filtered and the dark red filtrate was concentrated to approximately 0.5 ml. The viscous dark red solution was layered with pentane (2 ml) and stored at −35 °C freezer for 3 days, affording **5** as red-brown crystals. Yield: 0.19 g, 65%. Single crystals suitable for X-ray diffraction were grown from toluene solution at −35 °C. Note, this complex decomposes at room temperature within a few hours, even in crystalline form. Anal. Calcd for $C_{61}H_{77}N_6P_3Si_3U$: C 55.95; H 5.93; N 6.42%. Found: C 55.27; H 6.36; N 6.79%. ^{1}H NMR (C_6D_6, 298 K): δ −32.57 (br, 1 H), −30.66 (br, 3 H), −17.81 (br, 1 H), −15.56 (s, 18 H, SiCH_3), −4.68 (s, 3 H), −0.80-2.50 (m, br, 20 H), 3.20 (s, 3 H), 3.78 (s, 3 H), 4.50-5.60 (br, 6 H), 5.74 (s, 3 H), 5.99 (s, 6 H), 10.01 (br, 2 H), 11.62 (br, 3 H), 23.30 (s, 1 H), 25.30 (br, 3 H), 27.82 (s, 1 H), 47.58 (s, 1 H). ^{13}C NMR (C_6D_6, 298 K): δ 20.27, 45.13, 46.10, 56.38, 112.01, 112.48, 120.95, 121.11, 122.50, 122.91. ^{29}Si NMR (C_6D_6, 298 K): δ −163.89. ^{31}P NMR (C_6D_6, 298 K): δ −401.85 (s, U=C(*P*NSiMe$_3$Ph$_2$)$_2$). ATR-IR ν/cm^{-1}: 422 (m), 447 (m), 483 (m), 510 (s), 522 (s), 595 (s), 616 (w), 631 (m), 651 (m), 694 (s), 710 (s), 726 (m), 741 (s), 808 (s), 828 (s), 948 (w), 1000 (s), 1027 (w), 1066 (s), 1093 (w), 1107 (s), 1228 (s), 1241 (m), 1281 (w), 1305 (w), 1346 (s), 1383 (m), 1433 (m), 1466 (w), 1529 (w), 1607 (m), 2888 (w), 2939 (w).

Preparation of [U(BIPM^{TMS}){CH(Ph)(SiMe$_3$)}(CH$_2$SiMe$_3$)] (6)

Under N_2 atmosphere at −78 °C, [Li{CH(Ph)(SiMe$_3$)}(TMEDA)] (0.54 g, 1.90 mmol) in toluene (10 ml) was introduced dropwise into a stirring suspension of **1** (2.18 g, 2.00 mmol) in toluene (15 ml). The mixture was stirred at −78 °C for 30 minutes, and then stirred at room temperature for 1 hour. After this time the dark red solution was filtered, and the red filtrate, which contains **2** as the majority product, was subjected to next step. The solution was cooled to −78 °C and to this was added a solution of Me$_3$SiCH$_2$Li (0.17 g, 1.80 mmol) in toluene (10 ml) in a dropwise manner. The

dark red mixture was stirred at −78 °C for 15 minutes, and then stirred at room temperature for 1 hour. The mixture was then filtered, and the dark red filtrate was concentrated to a red viscous oil with an approximate volume of 0.5 ml. Pentane (3ml) was added and initially the red oil dissolved into pentane forming a red solution. After standing at room temperature overnight, red crystals appeared from the red solution. The mother liquor was removed and the red crystals were dried *in vacuo* to afford **6**. Yield: 0.55 g, 26%. Single crystals suitable for X-ray diffraction were grown from toluene/hexane mixture at −35 °C. Instability of **6** in solution prevented the acquisition of a reliable UV/Vis/NIR spectrum. Anal. Calcd for $C_{45}H_{64}N_2P_2Si_4U$: C 51.71; H 6.17; N 2.68%. Found: C 51.70; H 6.26; N 2.77%. ^{1}H NMR (C_6D_6, 298 K): δ −9.45 (s, 9 H, SiCH_3), −7.88 (s, 1 H, U-C*H*$SiMe_3$Ph), −3.59 (s, 9 H, SiCH_3), −2.20 (s, 9 H, SiCH_3), −1.39 (s, 2 H, UCH_2SiMe_3), −0.72 (s, 1 H), −0.07 (s, 1 H), 0.18 (s, 1 H), 0.26 (s, 1 H), 0.30 (s, 1 H), 3.68 (t, 2 H, $^3J_{HH}$ = 6.7 Hz, Ar*H*), 3.79 (t, 1 H, $^3J_{HH}$ = 6.8 Hz, Ar*H*), 4.62 (t, 1 H, $^3J_{HH}$ = 7.1 Hz, Ar*H*), 4.75 (br, 2 H), 4.95 (t, 2 H, $^3J_{HH}$ = 6.4 Hz, Ar*H*), 6.71 (s, 1 H), 7.23 (t, 1 H, , $^3J_{HH}$ = 7.3 Hz, Ar*H*), 7.71 (br, 1 H), 8.05 (t, 2 H, $^3J_{HH}$ = 7.0 Hz, Ar*H*), 10.03 (t, 1 H, $^3J_{HH}$ = 7.2 Hz, Ar*H*), 11.64 (t, 2 H, $^3J_{HH}$ = 6.5 Hz, Ar*H*), 11.80 (s, 9 H, SiMe_3), 13.56 (s, br, 2 H), 22.30 (s, 2 H). ^{31}P NMR (C_6D_6, 298 K): δ −584.11 (s, U=C(*P*N$SiMe_3Ph_2$)$_2$), -434.21 (s, U=C(*P*N$SiMe_3Ph_2$)$_2$). ATR-IR ν/cm^{-1}: 418 (s), 460 (m), 492 (s), 511 (s), 524 (m), 554 (s), 603 (s), 618 (w), 646 (s), 671 (m), 692 (s), 710 (s), 739 (m), 749 (m), 768 (m), 825 (s), 885 (w), 1017 (s), 1044 (m), 1107 (s), 1152 (w), 1179 (w), 1218 (m), 1248 (s), 1435 (m), 1475 (m), 1588 (w), 2891 (w), 2947 (w), 3057 (w).

Preparation of [U(BIPM^{TMS})(NC_5H_3-4-NMe_2)(CH_2SiMe_3)] (7)

At −78 °C, toluene (20 ml) was added to a stirring solid mixture of **6** (1.05 g, 1.00 mmol) and DMAP (0.12 g, 1.00 mmol). The mixture was stirred at −78 °C for 15 minutes and then stirred at room temperature for 24 hours. The red mixture was filtered, and the red filtrate was concentrated to approximately 1.5 ml and stored at −35 °C for 3 days, affording **7** as red-brown crystals. Yield: 0.32 g, 32%. Single crystals suitable for X-ray diffraction were grown from toluene/hexane mixture

at −35 °C. Anal. Calcd for $C_{42}H_{58}N_4P_2Si_3U$: C 50.29; H 5.83; N 5.59%. Found: C 51.57; H 5.66; N 5.10%. ^{1}H NMR (C_6D_6, 298 K): δ −59.55 (br, 2 H, U-CH_2SiMe$_3$), −24.36 (s, 9 H, SiCH_3), −22.32 (s, 1 H, U-CH(SiMe$_3$)(Ph)), −16.03 (s, 9 H, SiCH_3), −2.19 (s, 3 H), 0.31 (s, 3 H), 0.98 (s, 2 H), 4.76 (s, 9 H, SiCH_3), 5.22 (s, 3 H), 6.45 (br, 4 H), 7.75 (br, 1.5 H), 8.43 (br, 1.5 H), 9.52 (s, 3 H), 11.98 (s, 2 H), 14.29 (s, 2 H), 18.70 (s, 2 H), 19.89 (s, 1 H), 20.14 (s, 9 H, SiCH_3), 27.96 (s, 1 H), 31.93 (s, 2 H), 56.83 (s, 1 H). ^{31}P NMR (C_6D_6, 298 K): δ −530.53 (br, U=C(PNSiMe$_3$Ph$_2$)$_2$), -350.94 (br, U=C(PNSiMe$_3$Ph$_2$)$_2$). ^{29}Si NMR (C_6D_6, 298 K): δ −118.86. ATR-IR ν/cm^{-1}: 412 (s), 447 (s), 483 (s), 502 (s), 544 (m), 600 (s), 645 (m), 692 (m), 710 (s), 730 (s), 762 (s), 828 (m), 987 (s), 1001 (m), 1042 (w), 1105 (m), 1153 (m), 1178 (s), 1243 (m), 1365 (m), 1433 (w), 1475 (w), 1494 (w), 1580 (w), 2946 (w).

Reaction of [U{C(SiMe$_3$)(PPh$_2$)}(BIPM^{TMS})(μ-Cl)Li(TMEDA)(μ-TMEDA)$_{0.5}$]$_2$ (3) with PhCHO

Benzaldehyde (0.006 g, 0.06 mmol) was added to a suspension of **3** (0.04 g, 0.02 mmol) in C_6D_6 (0.5 ml). The reaction mixture was shaken vigorously, forming a brown reaction mixture. The reaction mixture was stored under ambient temperature for 1 hour and then analysed by multi-nuclear NMR spectroscopy (^{1}H, ^{7}Li, and ^{31}P), which revealed quantitative conversion to (Me$_3$SiNPPh$_2$)$_2$C=C(H)Ph and (Ph$_2$P)(Me$_3$Si)C=C(H)(Ph). All spectroscopic data matched previously reported data.[4,5]

Reaction of [U{C(SiMe$_3$)(PPh$_2$)}(BIPM^{TMS})(μ-Cl)Li(TMEDA)(μ-TMEDA)$_{0.5}$]$_2$ (3) with 9-anthracenecarboxaldehyde (ArCHO)

9-Anthracenecarboxaldehyde (0.013 g, 0.06 mmol) was added to a suspension of **3** (0.04 g, 0.02 mmol) in C_6D_6 (0.5 ml). The reaction mixture was stored under ambient temperature for 1 hour and then analysed by multi-nuclear NMR spectroscopy (^{1}H, ^{7}Li, and ^{31}P), which revealed quantitative conversion to (Me$_3$SiNPPh$_2$)$_2$C=C(H)Ar and (Ph$_2$P)(Me$_3$Si)C=C(H)(Ar). All spectroscopic data matched previously reported data.[4,5]

Reaction of [U{C(SiMe$_3$)(PPh$_2$)}(BIPM^{TMS})(Cl)][Li(2,2,2-cryptand)] (4) with PhCHO

Benzaldehyde (0.004 g, 0.04 mmol) was added to a suspension of **4** (0.03 g, 0.02 mmol) in C_6D_6 (0.5 ml). The reaction mixture was shaken vigorously, forming a brownish yellow solution with large amount of grey precipitate. The reaction mixture was stored under ambient temperature for 30 minutes and then analysed by multi-nuclear NMR spectroscopy (^{1}H, ^{7}Li, and ^{31}P), which revealed quantitative conversion to $(Me_3SiNPPh_2)_2C{=}C(H)Ph$ and $(Ph_2P)(Me_3Si)C{=}C(H)(Ph)$. All spectroscopic data matched previously reported data.[4,5]

Reaction of [U{C(SiMe$_3$)(PPh$_2$)}(BIPM^{TMS})(Cl)][Li(2,2,2-cryptand)] (4) with 9-anthracenecarboxaldehyde (ArCHO)

9-Anthracenecarboxaldehyde (0.008 g, 0.04 mmol) was added to a suspension of **4** (0.03 g, 0.02 mmol) in C_6D_6 (0.5 ml). The reaction mixture was shaken vigorously, forming a red reaction mixture. The reaction mixture was stored under ambient temperature for 16 hours and analysed by multi-nuclear NMR spectroscopy (^{1}H, ^{7}Li, and ^{31}P), which revealed that no reaction occurred. The reaction mixture was heated to 60 °C for 12 hours and analysed by multi-nuclear NMR spectroscopy (^{1}H, ^{31}P, and ^{7}Li), which indicated full conversion to $(Me_3SiNPPh_2)_2C{=}C(H)Ar$ and $(Ph_2P)(Me_3Si)C{=}C(H)(Ar)$. All spectroscopic data matched previously reported data.[4,5]

Reaction of [U{C(SiMe$_3$)(PPh$_2$)}(BIPM^{TMS})(DMAP)$_2$] (5) with PhCHO

Benzaldehyde (0.004 g, 0.04 mmol) was added to a suspension of **5** (0.03 g, 0.02 mmol) in C_6D_6 (0.5 ml). The reaction mixture was shaken vigorously, forming a brownish red solution with large amount of grey precipitate. The reaction mixture was stored under ambient temperature for 12 hours and analysed by multi-nuclear NMR spectroscopy (^{1}H and ^{31}P), which revealed full conversion to $(Me_3SiNPPh_2)_2C{=}C(H)Ph$ and $(Ph_2P)(Me_3Si)C{=}C(H)(Ph)$. All spectroscopic data matched previously reported data.[4,5]

Reaction of [U{C(SiMe$_3$)(PPh$_2$)}(BIPM^{TMS})(DMAP)$_2$] (5) with 9-anthracenecarboxaldehyde (ArCHO)

9-Anthracenecarboxaldehyde (0.008 g, 0.04 mmol) was added to a suspension of **4** (0.03 g, 0.02 mmol) in C_6D_6 (0.5 ml). The reaction mixture was shaken vigorously, forming a brownish red reaction mixture. The reaction mixture was stored under ambient temperature for 16 hours and analysed by multi-nuclear NMR spectroscopy (^{1}H and ^{31}P), which revealed that no reaction occurred. The reaction mixture was heated to 60 °C for 24 hours and analysed by multi-nuclear NMR spectroscopy (^{1}H, ^{31}P, and ^{7}Li), which indicated full conversion to $(Me_3SiNPPh_2)_2C$=C(H)Ar and $(Ph_2P)(Me_3Si)C$=C(H)(Ar). All spectroscopic data matched previously reported data.[4,5]

Preparation of [U{C(SiMe$_3$)(Ph$_2$PCPhCPh)}(BIPM^{TMS})] (8)

Diphenylacetylene (0.05 g, 0.26 mmol) in toluene (3 ml) was added to a suspension of **3** (0.35 g, 0.13 mmol) in toluene (3 ml), leading to the rapid formation of a dark red solution. The solution was stirred for one hour at ambient temperature, before being filtered and concentrated to approximately 2 ml. Slow evaporation of solvent was carried out in a glovebox, leading to the formation of a large crop of dark red crystals. Yield: 0.25 g, 76%. Single crystals suitable for X-ray diffraction were grown from toluene solution with slow evaporation of the solvent. Anal. Calcd for $C_{62}H_{71}N_2P_3Si_3U$: C 58.92; H 5.43; N 2.25%. Found: C 58.06; H 5.41; N 2.21%. ^{1}H NMR (C_6D_6, 298 K): δ −36.13 (br, 18 H, SiCH_3), −27.58 (br, 9 H, SiCH_3), −5.34 (br, 3 H), −3.11 (br, 3 H), −1.41 (s, 2 H), 1.98 (br, 1 H), 3.65 (br, 1 H), 7.83 (br, 1 H), 8.06 (br, 2 H), 8.25 (br, 1 H), 13.00 (br, 1 H), 15.28 (br, 1 H), 15.45 (br, 2 H), 18.34 (br, 2H), 42.01 (br, 2 H), 43.80 (br, 1 H). ^{31}P NMR (C_6D_6, 298 K): δ −89.27 (br, U=C(PNSiMe$_3$Ph$_2$)$_2$), 281.21 (br, U=C(SiMe$_3$)[*P*Ph$_2$-(Ph)C=C(Ph)]). ^{29}Si NMR (C_6D_6, 298 K): δ −109.88. ATR-IR ν/cm^{-1}: 447 (s), 483 (s), 503 (s), 547 (s), 602 (s), 641 (s), 666 (s), 691 (s), 742 (s), 775 (s), 828 (m), 999 (s), 1026 (s), 1055 (s), 1099 (s), 1245 (w), 1434 (s), 1479 (s), 1588 (s), 2892 (m), 2946 (m), 3016 (m), 3050 (m).

Single Crystal X-ray Crystallography

Crystals were examined using either a) an Agilent Supernova diffractometer, equipped with Eos CCD area detector and a Microfocus source with Mo Kα radiation (λ = 0.71073 Å) or b) a Rigaku FR-X diffractometer, equipped with a HyPix 6000HE photon counting pixel array detector with mirror-monochromated Mo Kα (λ = 0.71073 Å) or Cu Kα (λ = 1.5418 Å) radiation. Intensities were integrated from a sphere of data recorded on narrow (0.5 or 1.0°) frames by *ω* rotation. Cell parameters were refined from the observed positions of all strong reflections in each data set. A Gaussian grid face-indexed was applied in each case.[6] The structures were solved by direct methods using either SHELXS or SHELXT, or iteratively with SUPERFLIP[7,8] and the dataset was refined by full-matrix least-squares on all unique F^2 values, with anisotropic displacement parameters for all non-hydrogen atoms, and with constrained riding hydrogen geometries; U_{iso}(H) was set at 1.2 (1.5 for methyl groups) times U_{eq} of the parent atom. The largest features in final difference syntheses were close to heavy atoms and were of no chemical significance. CrysAlisPro[9] was used for control and integration, and SHELX[7,8] and OLEX2[10] were employed for structure solution and refinement. ORTEP-3[11] and POV-Ray[12] were employed for molecular graphics.

Computational Details

Unrestricted geometry optimisations were performed using coordinates derived from crystal structures as the start-points. No constraints were imposed on the structures during the geometry optimisations. The calculations were performed using the Amsterdam Density Functional (ADF) suite version 2012.01.[13,14] The DFT geometry optimisations employed Slater type orbital (STO) triple-ζ-plus polarisation all-electron basis sets (from the ZORA/TZP database of the ADF suite). Scalar relativistic approaches were used within the ZORA Hamiltonian for the inclusion of relativistic effects and the local density approximation (LDA) with the correlation potential due to Vosko *et al* was used in all of the calculations.[15] Gradient corrections were performed using the functionals of Becke and Perdew.[16,17] MOLEKEL[18] was used to prepare the three-dimensional plots

of the electron density. Natural Bond Order (NBO) analyses were carried out with NBO 5.0.[19] The Atoms in Molecules analysis[20,21] was carried out with Xaim-1.0.[22]

Single Crystal X-ray Diffraction Structures

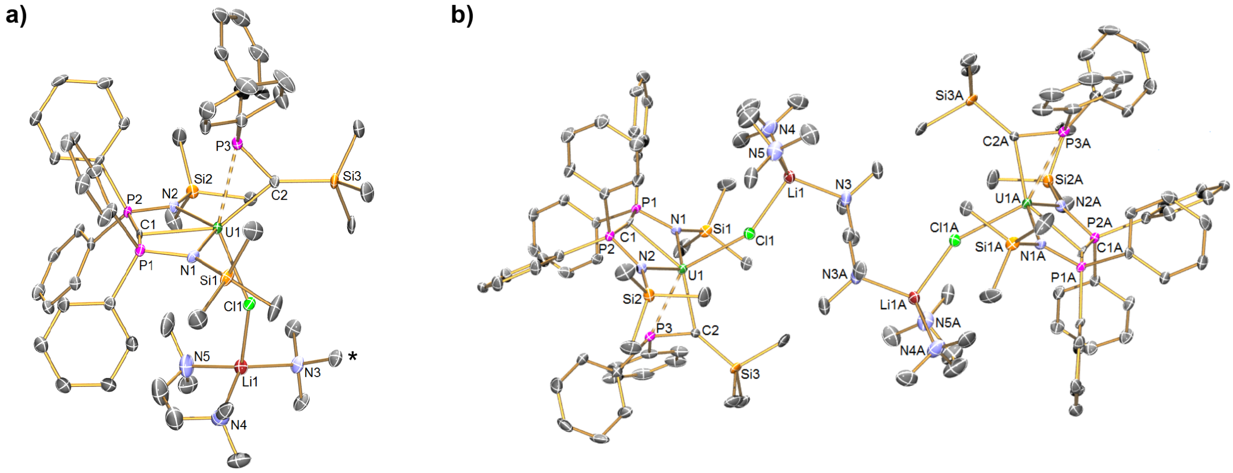

Figure S1. Molecular structure of a) the crystallographic asymmetric unit and b) the whole molecule of crystalline $[U\{C(SiMe_3)(PPh_2)\}(BIPM^{TMS})(\mu\text{-}Cl)Li(TMEDA)(\mu\text{-}TMEDA)_{0.5}]_2$ (**3**) at 150 K with 40% probability ellipsoids. The weak U=C-P interaction is represented by a dashed-bond between uranium and phosphorus. The * indicates the methylene carbon of the bridging TMEDA ligand.

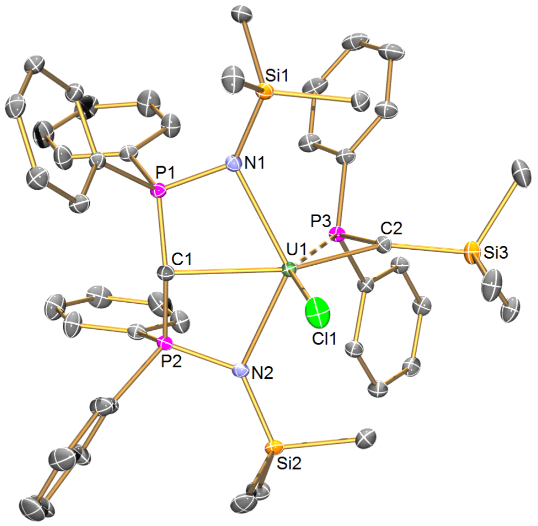

Figure S2. Molecular structure of the anion component of crystalline [U{C(SiMe$_3$)(PPh$_2$)}(BIPM^{TMS})(Cl)][Li(2,2,2-cryptand)] (**4**) at 150 K with 40% probability ellipsoids. Hydrogen atoms, the unremarkable [Li(2,2,2-cryptand)]$^+$ cation, and lattice solvent are omitted for clarity. Two units of **4** crystallize in the crystallographic asymmetric unit but due to their similarity only one is highlighted. The weak U=C-P interaction is represented by a dashed-bond between uranium and phosphorus.

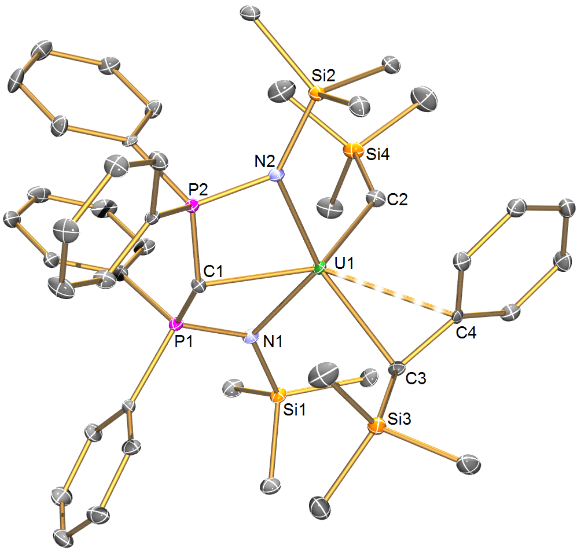

Figure S3. Molecular structure of the full molecule of crystalline [U(BIPM^{TMS}){CH(Ph)(SiMe$_3$)}(CH$_2$SiMe$_3$)] (**6**) at 150 K with 40% probability ellipsoids. Hydrogen atoms, minor disorder components, and lattice solvent are omitted for clarity. A close U···C$_{ipso}$ interaction is represented by a dashed bond.

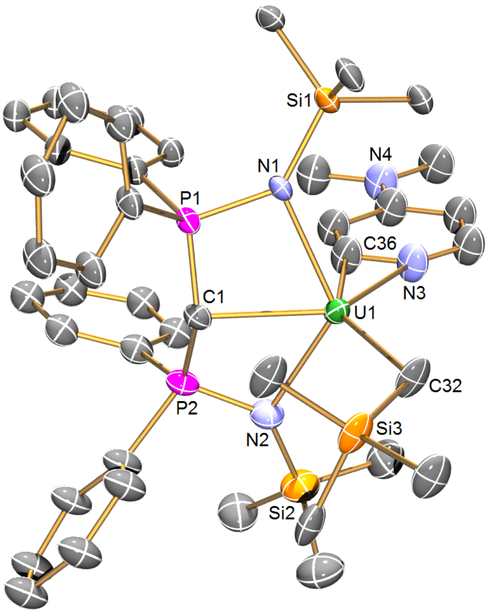

Figure S4. Molecular structure of the full molecule of crystalline [U(BIPM^{TMS})(NC_5H_3-4-NMe_2)(CH_2SiMe_3)] (**7**) at 150 K with 40% probability ellipsoids. Hydrogen atoms, minor disorder components, and lattice solvent are omitted for clarity.

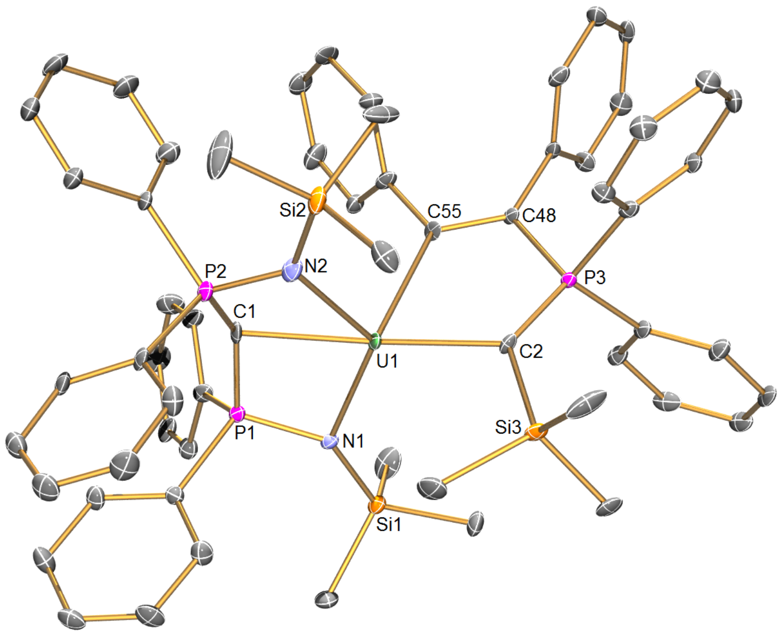

Figure S5. Molecular structure of [U{C($SiMe_3$)(Ph_2PCPhCPh)}(BIPM^{TMS})] (**8**) at 150 K with 40% probability ellipsoids. Hydrogen atoms are omitted for clarity.

NMR Spectra

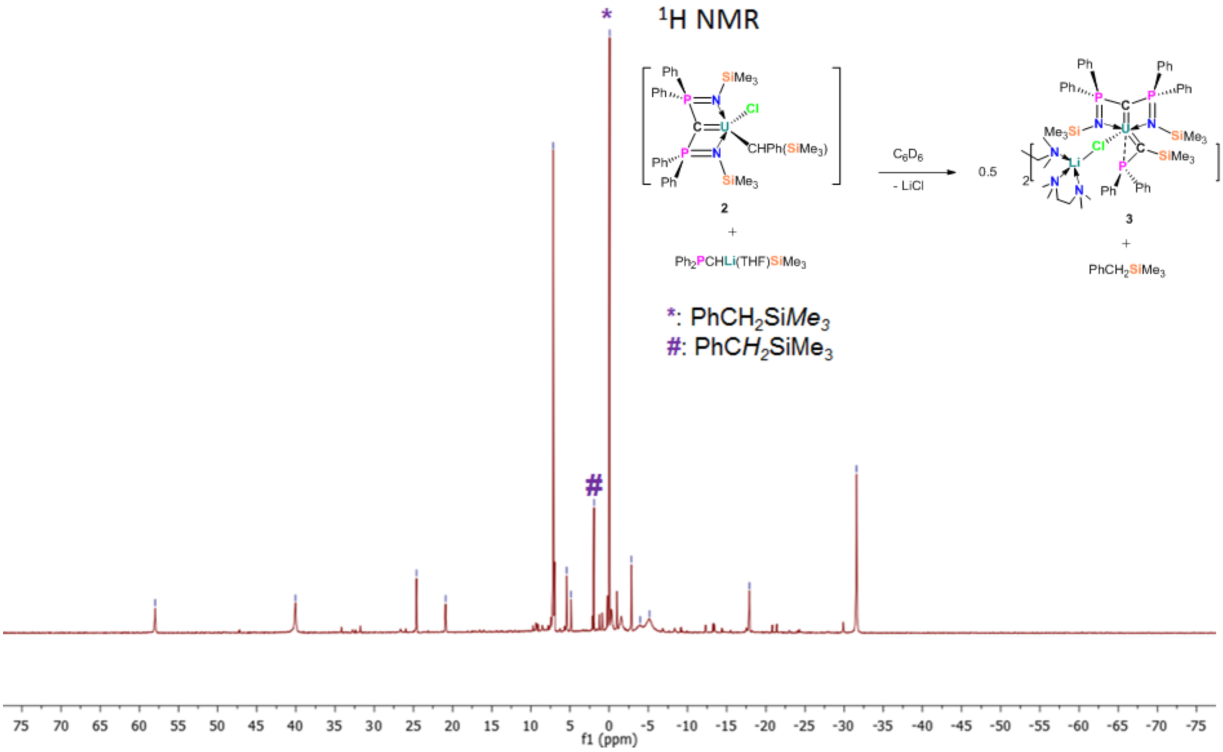

Figure S6. ^{1}H NMR spectrum of **3** and $PhCH_2SiMe_3$, from reaction between **2** and 1 equivalent of $[Li\{CH(SiMe_3)(PPh_2)\}(THF)]$ in C_6D_6.

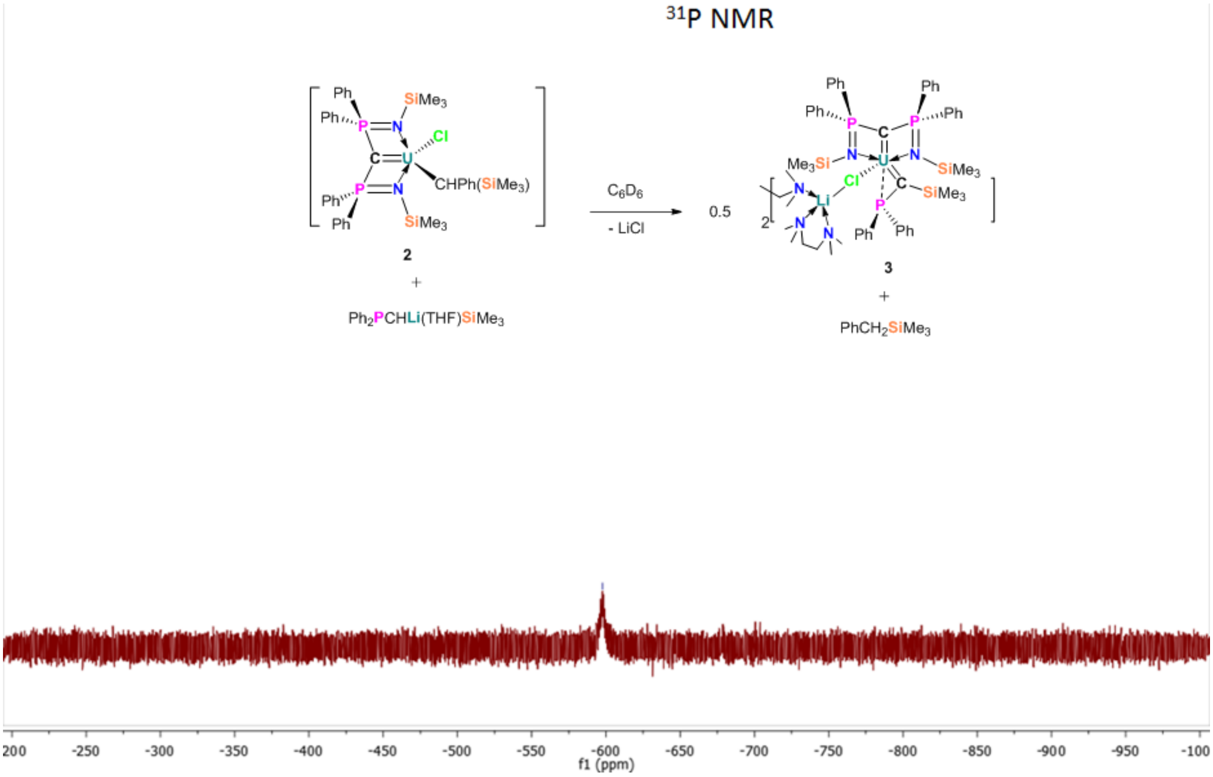

Figure S7. ^{31}P NMR spectrum of **3** and $PhCH_2SiMe_3$, from reaction between **2** and 1 equivalent of $[Li\{CH(SiMe_3)(PPh_2)\}(THF)]$ in C_6D_6.

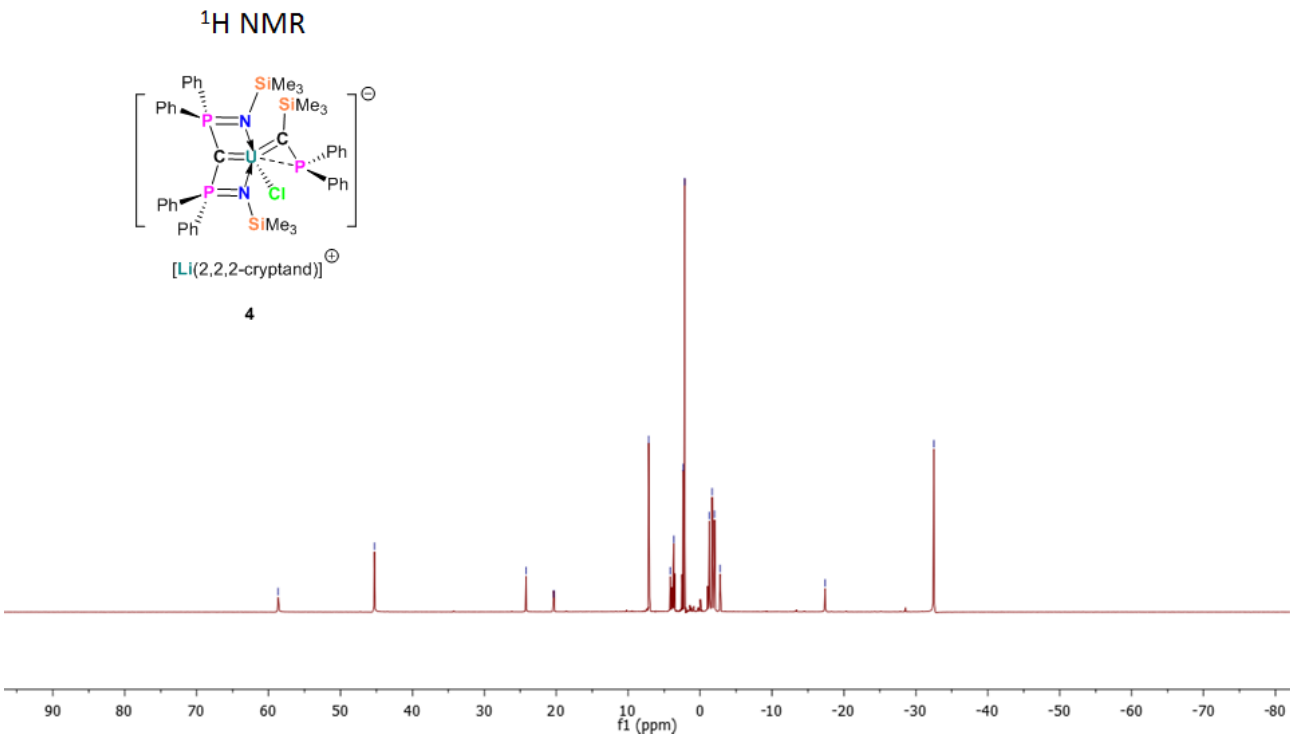

Figure S8. ^{1}H NMR spectrum of **4**, from reaction between **3** and 1 equivalent of 2,2,2-cryptand in C_6D_6.

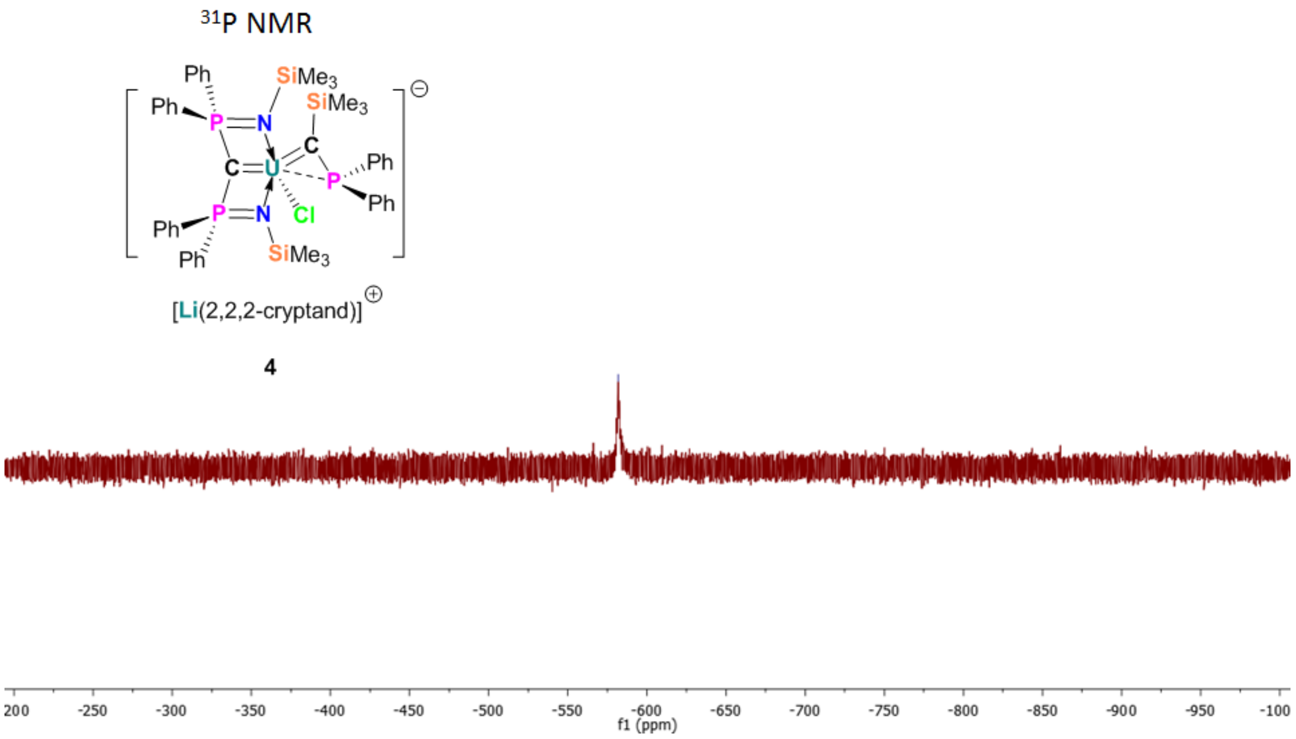

Figure S9. ^{31}P NMR spectrum of **4**, from reaction between **3** and 1 equivalent of 2,2,2-cryptand in C_6D_6.

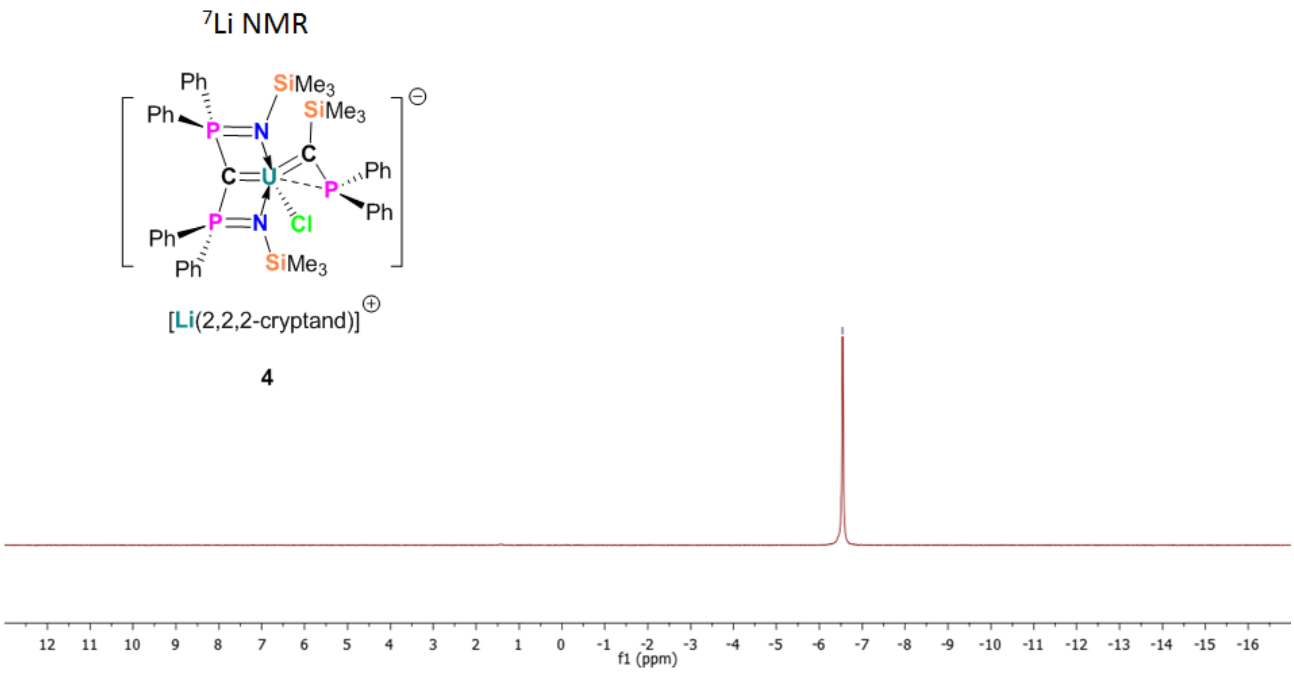

Figure S10. ^{7}Li NMR spectrum of **4**, from reaction between **3** and 1 equivalent of 2,2,2-cryptand in C_6D_6.

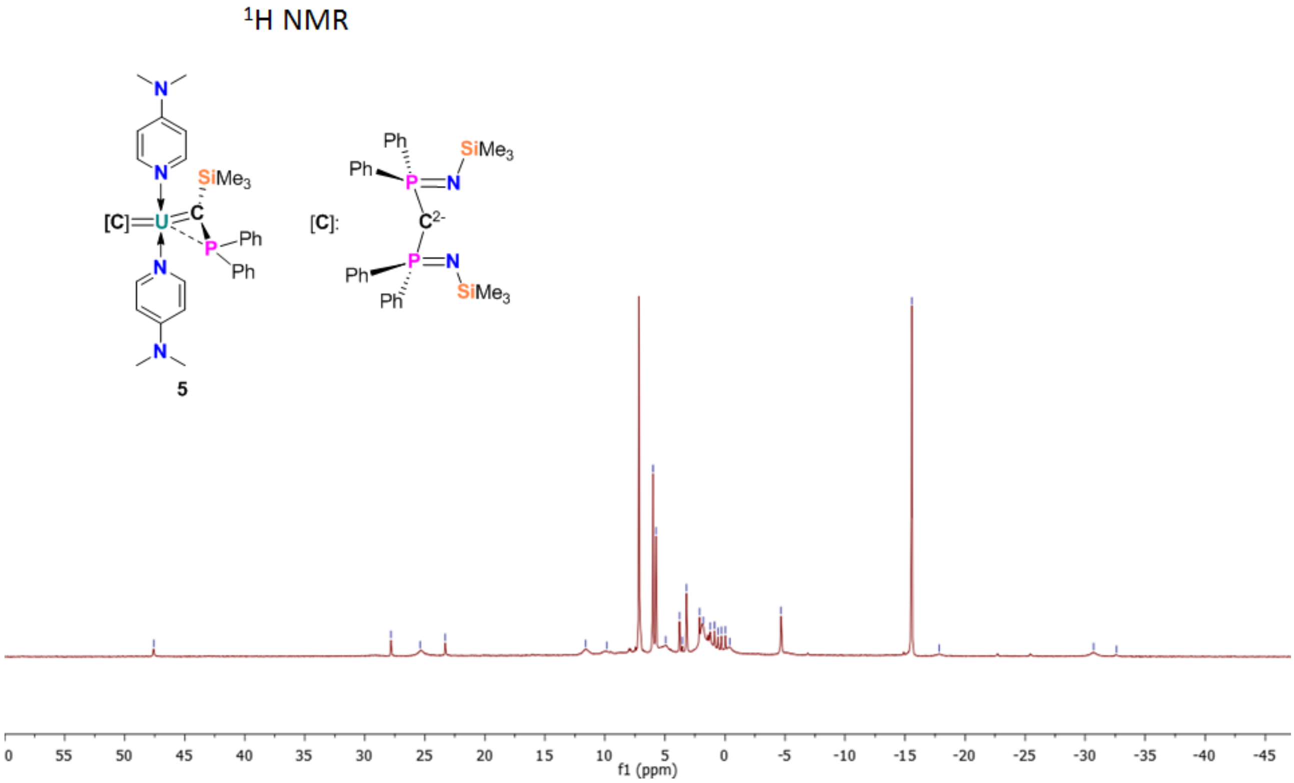

Figure S11. ^{1}H NMR spectrum of **5**.

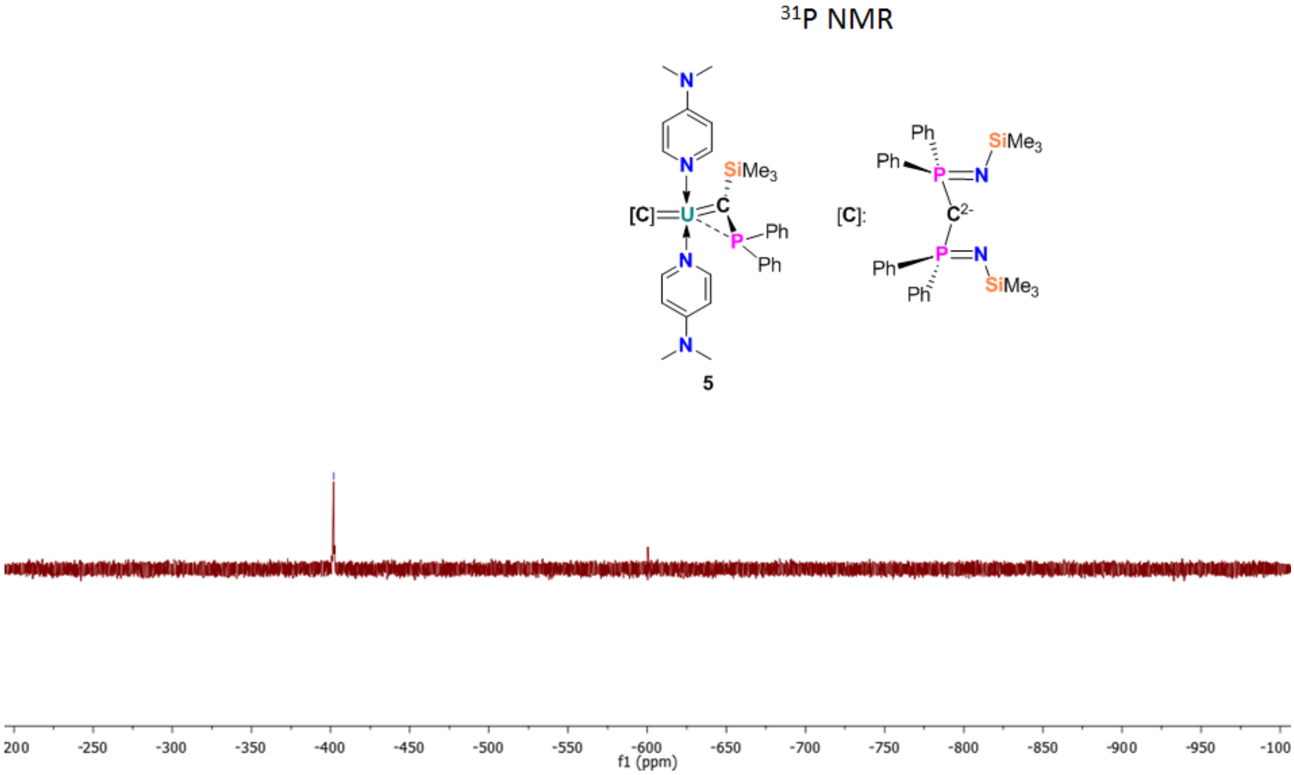

Figure S12. ^{31}P NMR spectrum of **5**. The line at –600 ppm is of no meaningful integration area, and is the spectrometer background noise, which persists at the central point of a spectrum.

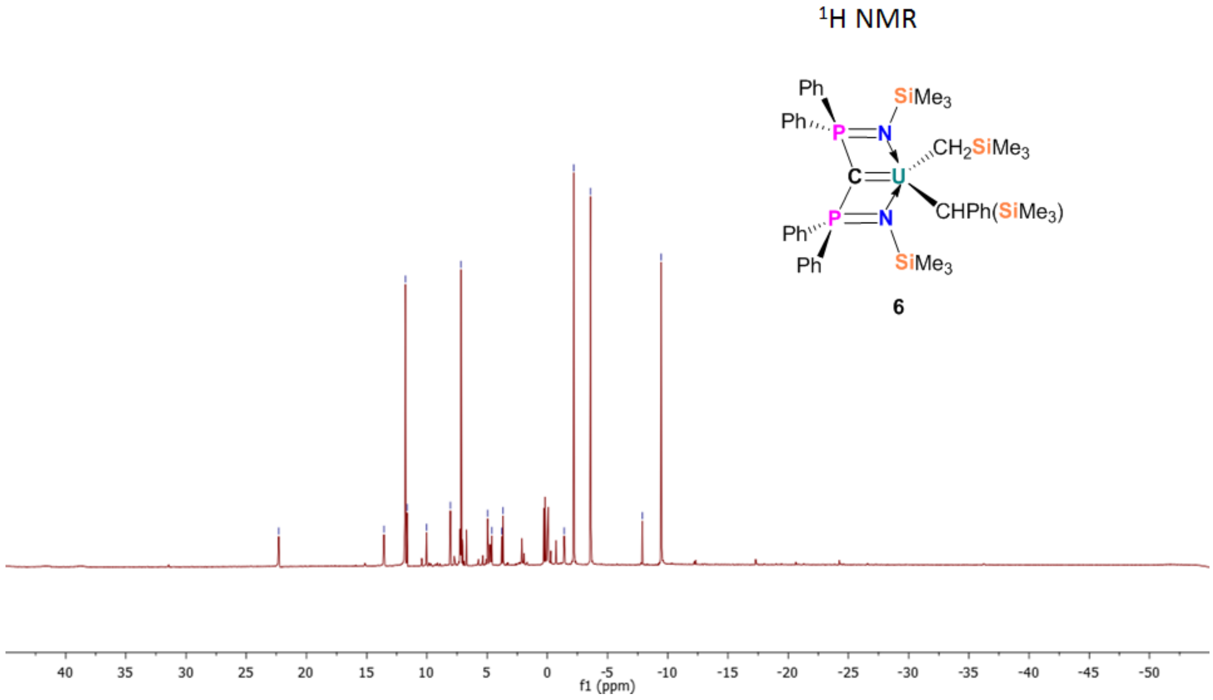

Figure S13. ^{1}H NMR spectrum of **6**.

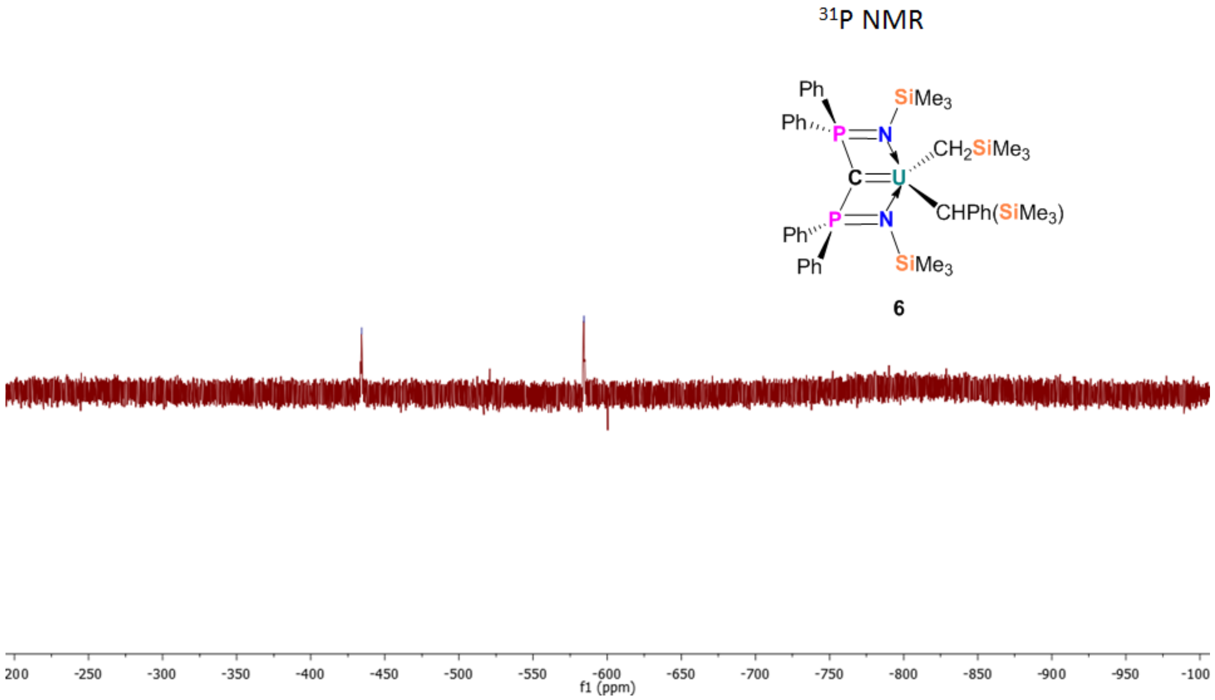

Figure S14. ^{31}P NMR spectrum of **6**. The line at −600 ppm is of no meaningful integration area, and is the spectrometer background noise, which persists at the central point of a spectrum.

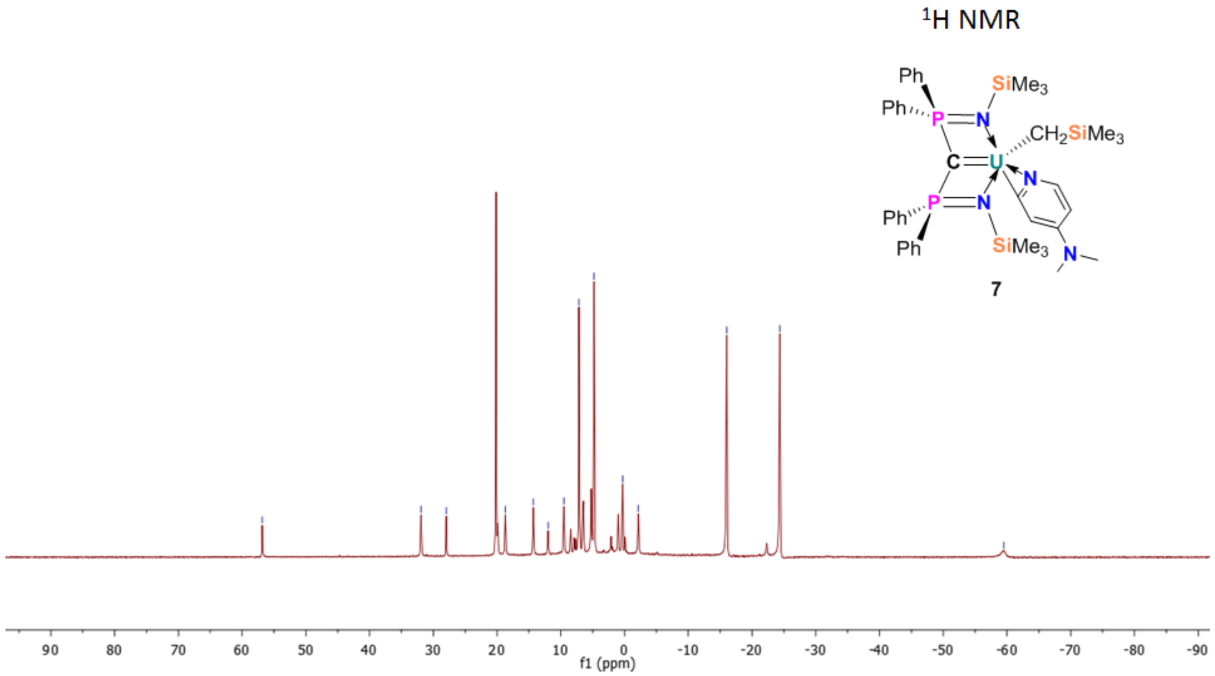

Figure S15. ^{1}H NMR spectrum of **7**.

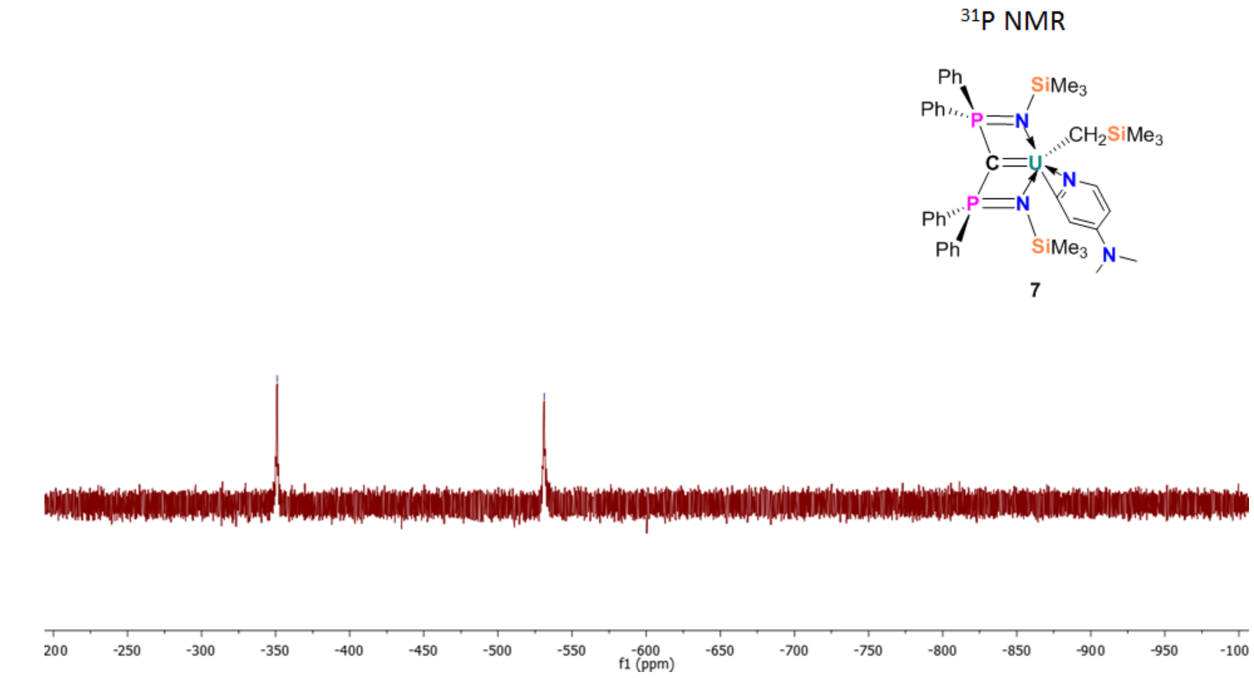

Figure S16. ^{31}P NMR spectrum of **7**.

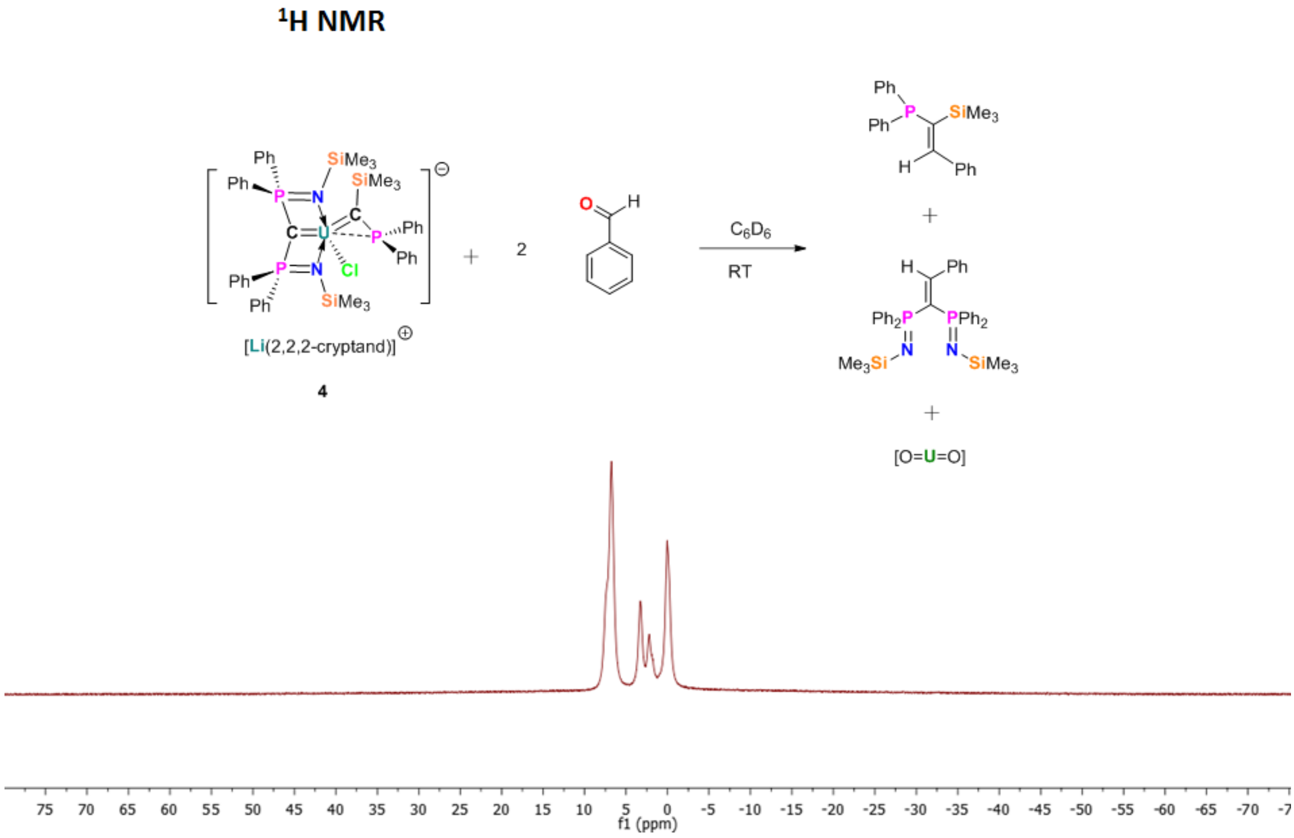

Figure S17. ^{1}H NMR reaction of **4** with 2 equivalents of PhCHO.

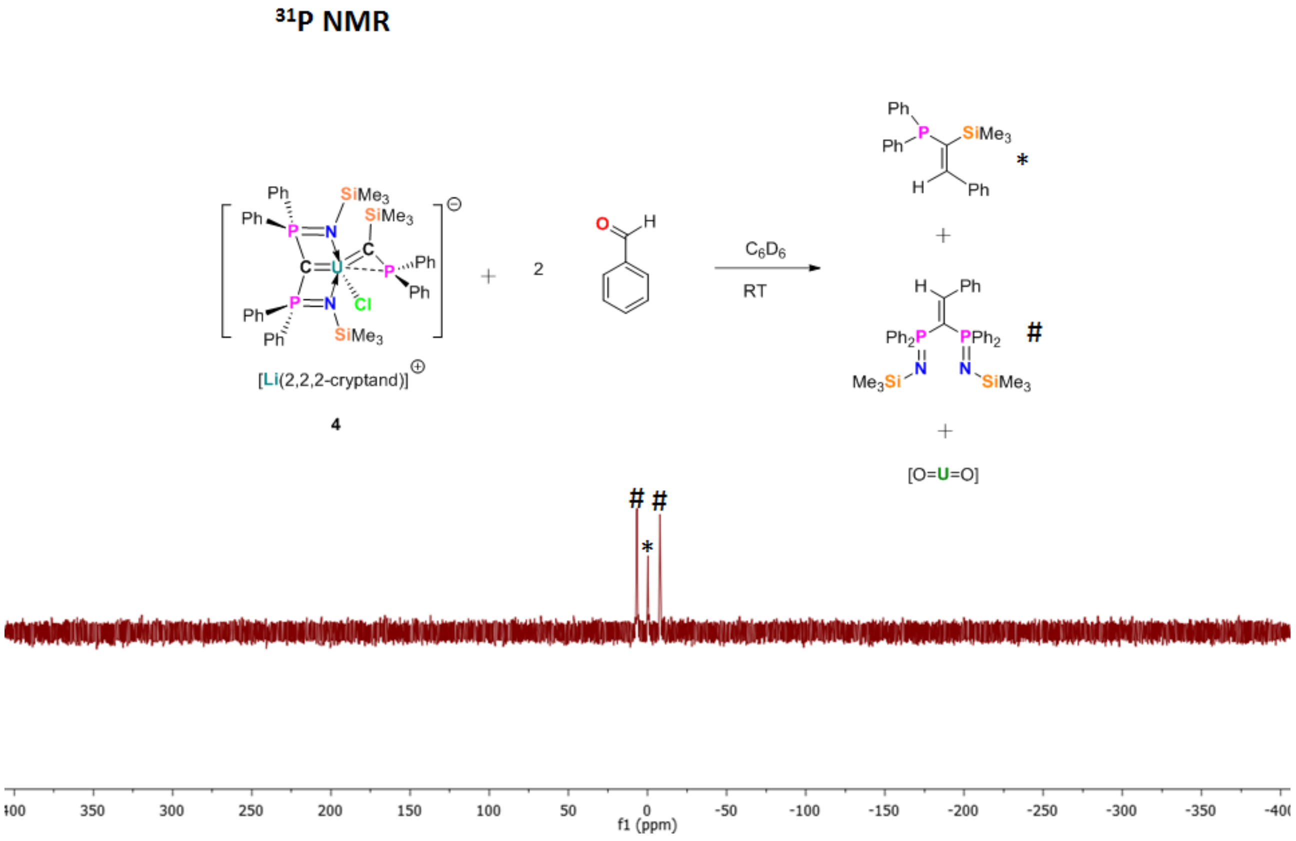

Figure S18. ^{31}P NMR reaction of **4** with 2 equivalents of PhCHO.

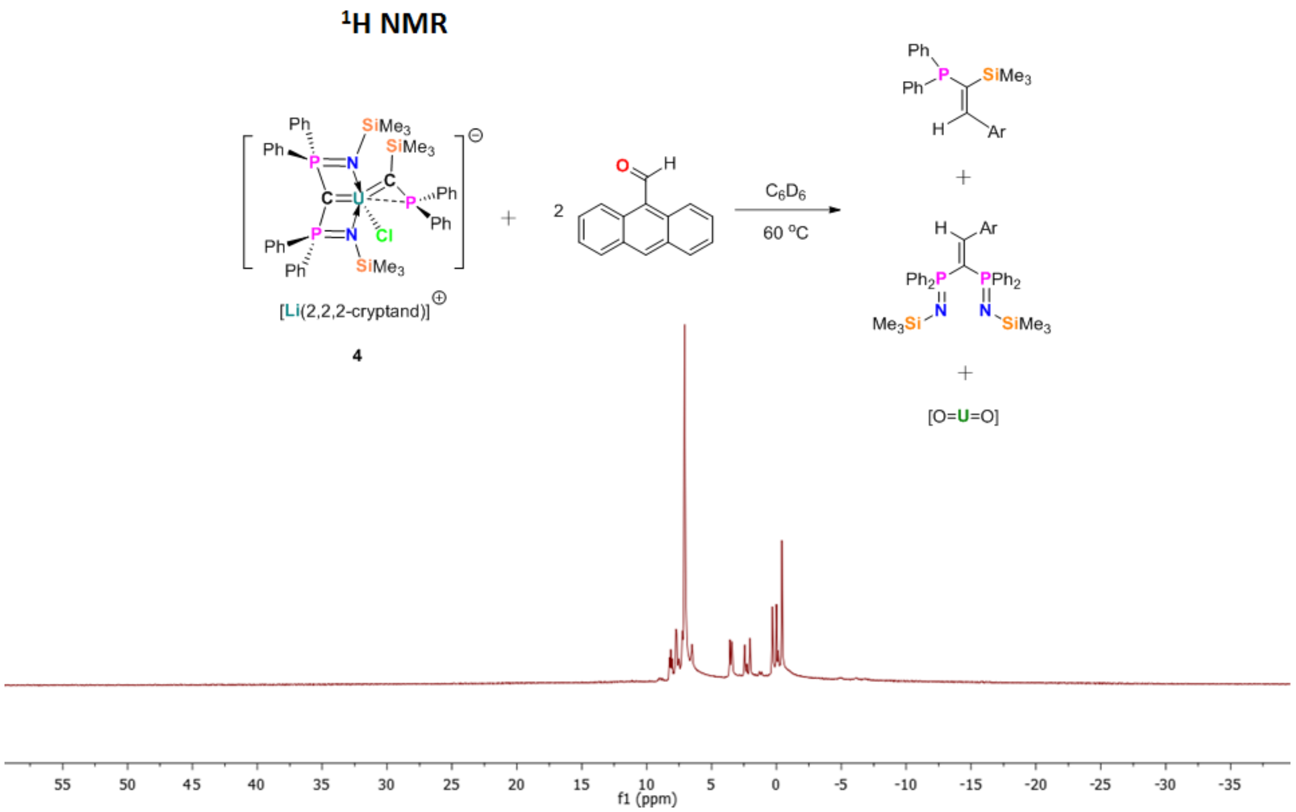

Figure S19. ^{1}H NMR reaction of **4** with 2 equivalents of 9-anthracenecarboxaldehyde.

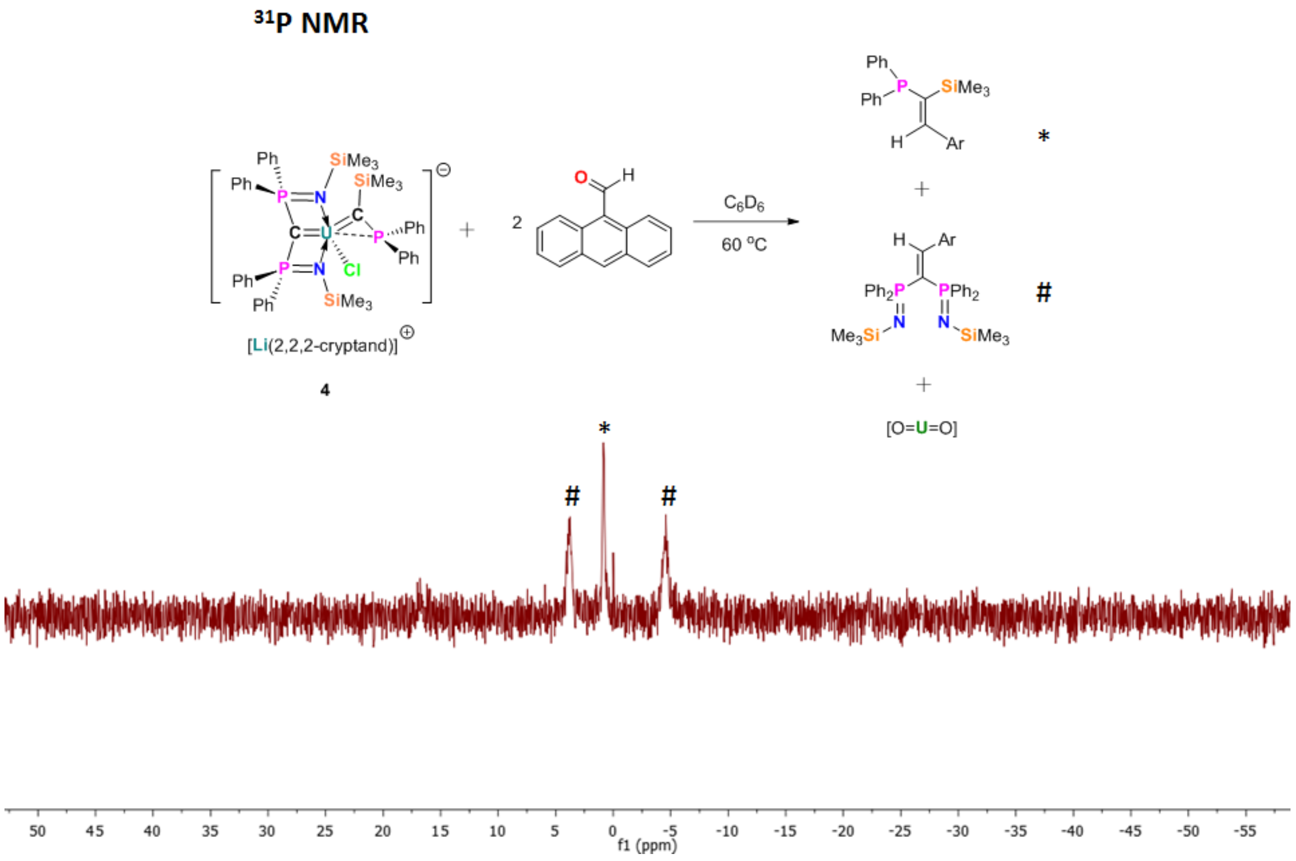

Figure S20. ^{31}P NMR reaction of **4** with 2 equivalents of 9-anthracenecarboxaldehyde.

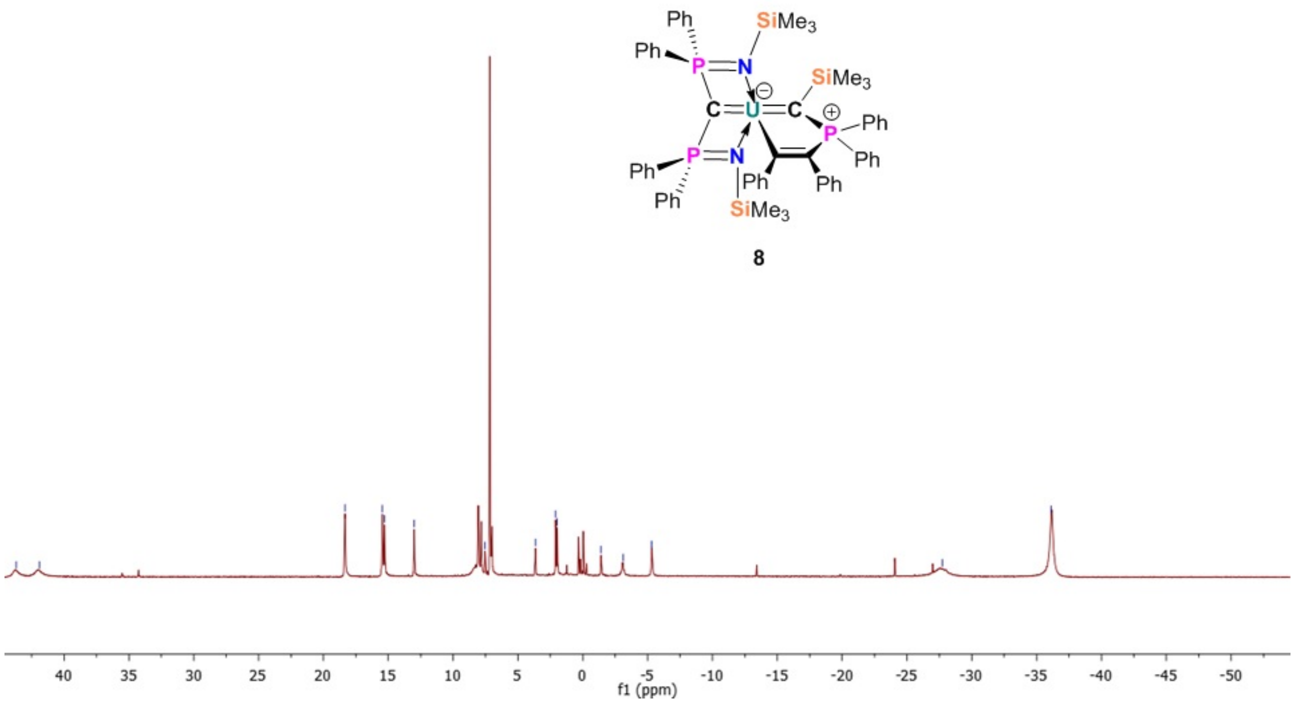

Figure S21. ^{1}H NMR spectrum of **8**.

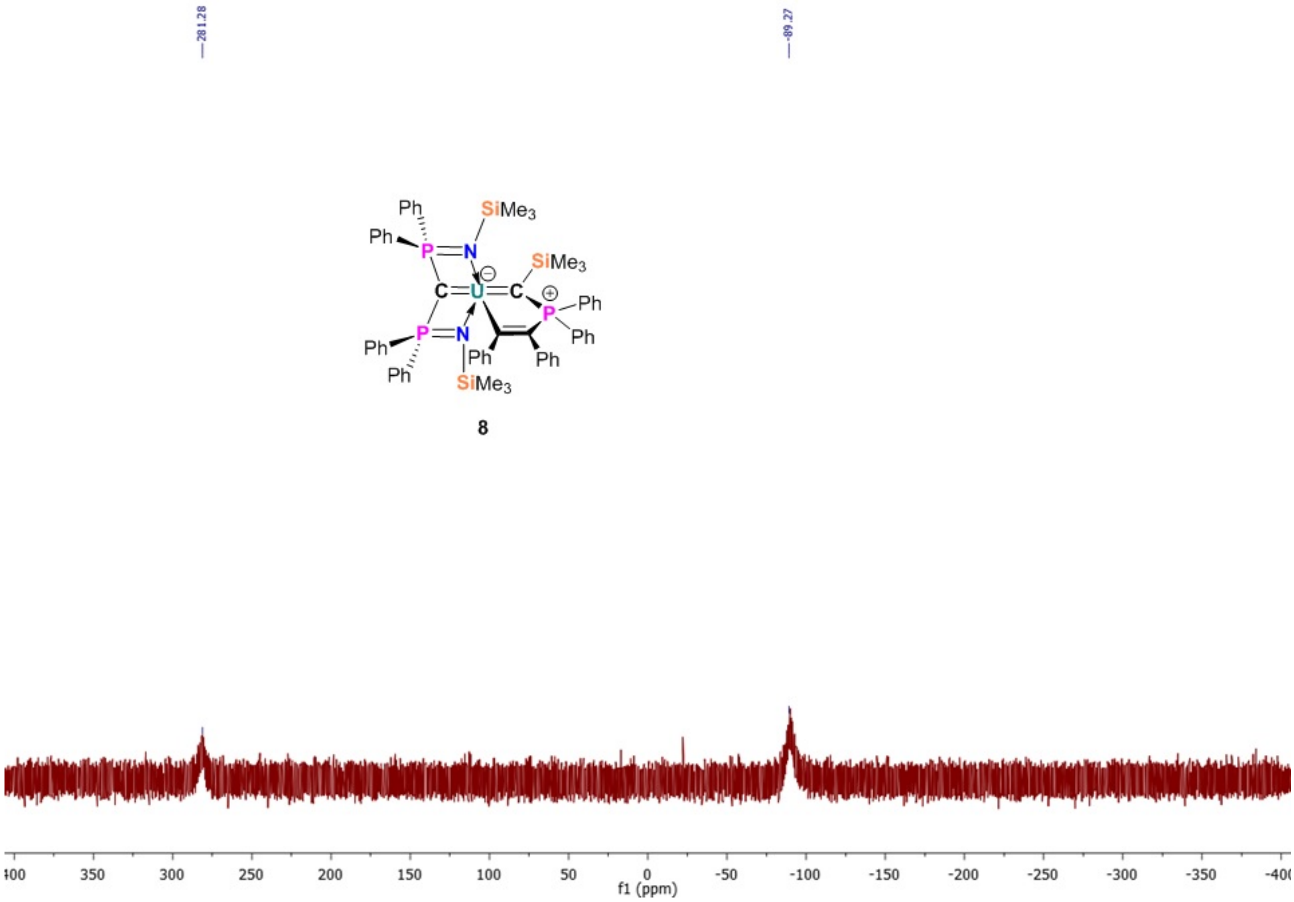

Figure S22. ^{31}P NMR spectrum of **8**.

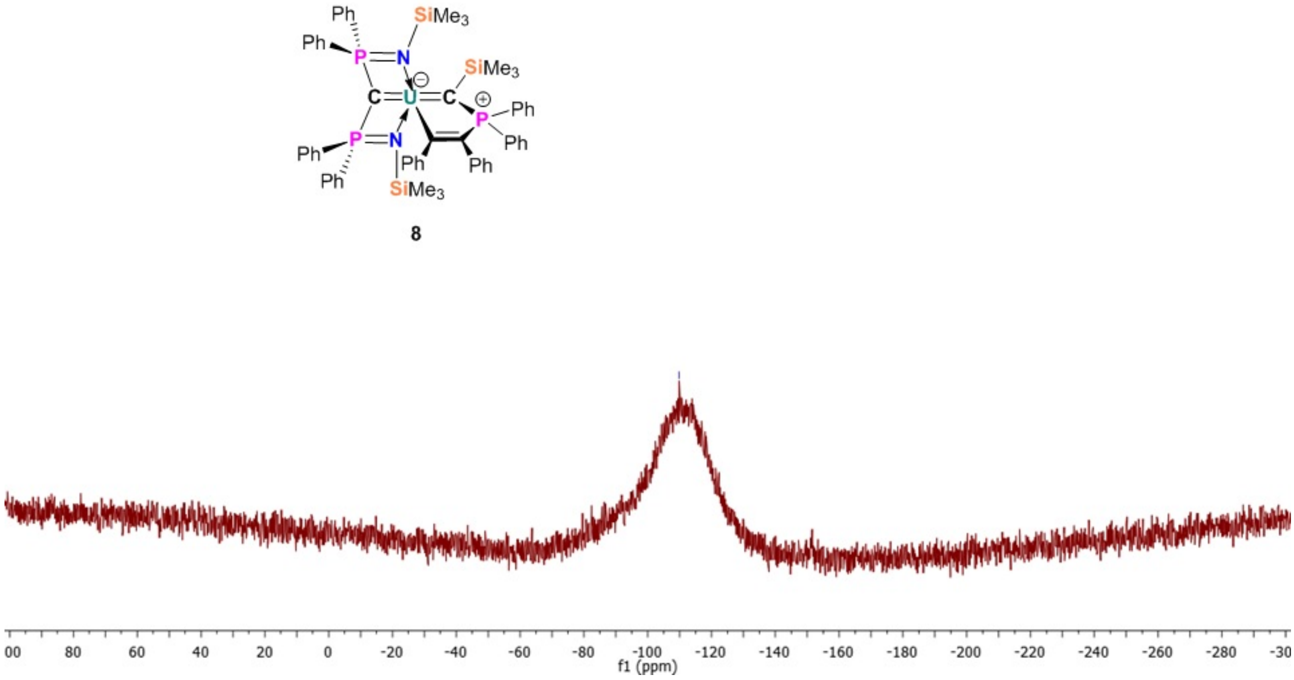

Figure S23. ^{29}Si NMR spectrum of **8**.

UV/Vis/NIR Spectra

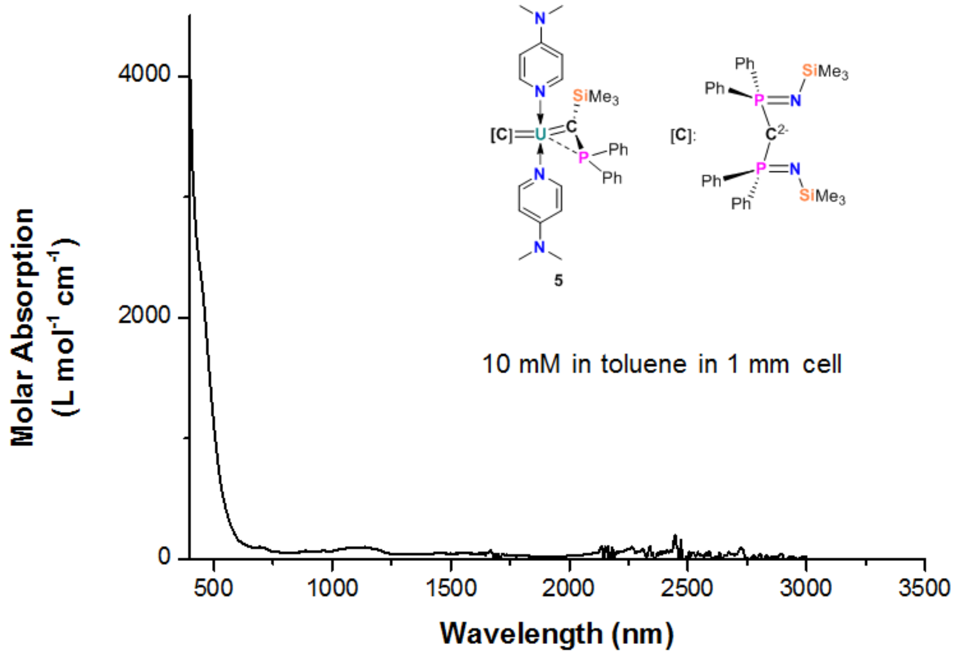

Figure S24. UV/Vis spectrum of **5**.

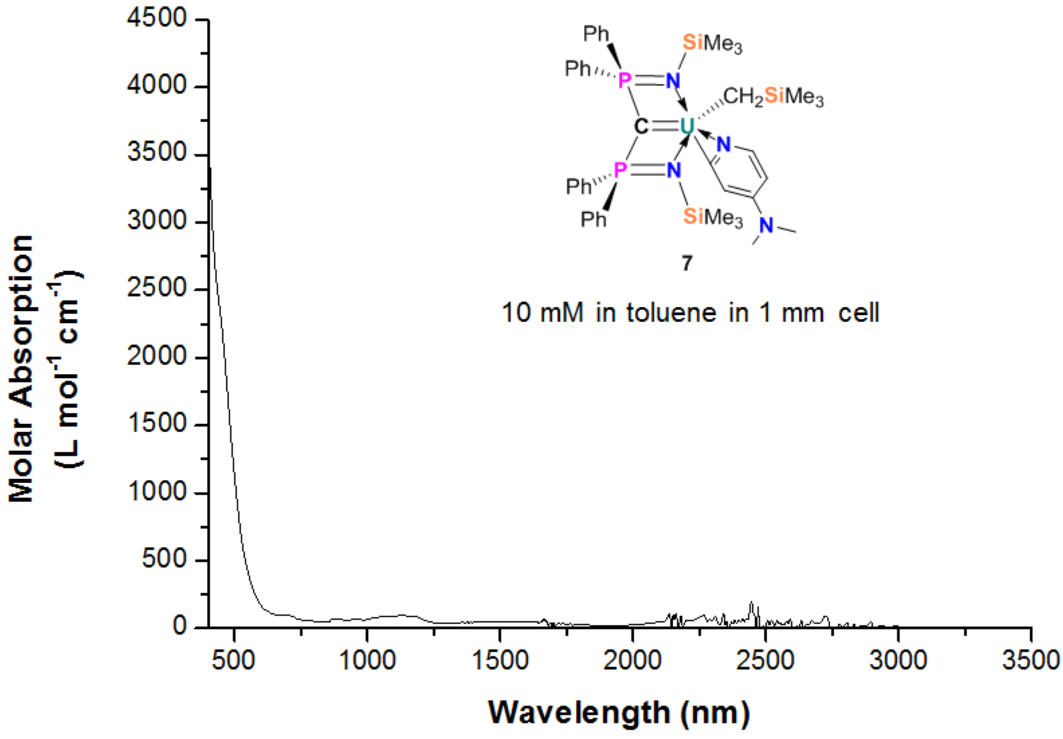

Figure S25. UV/Vis spectrum of **7**.

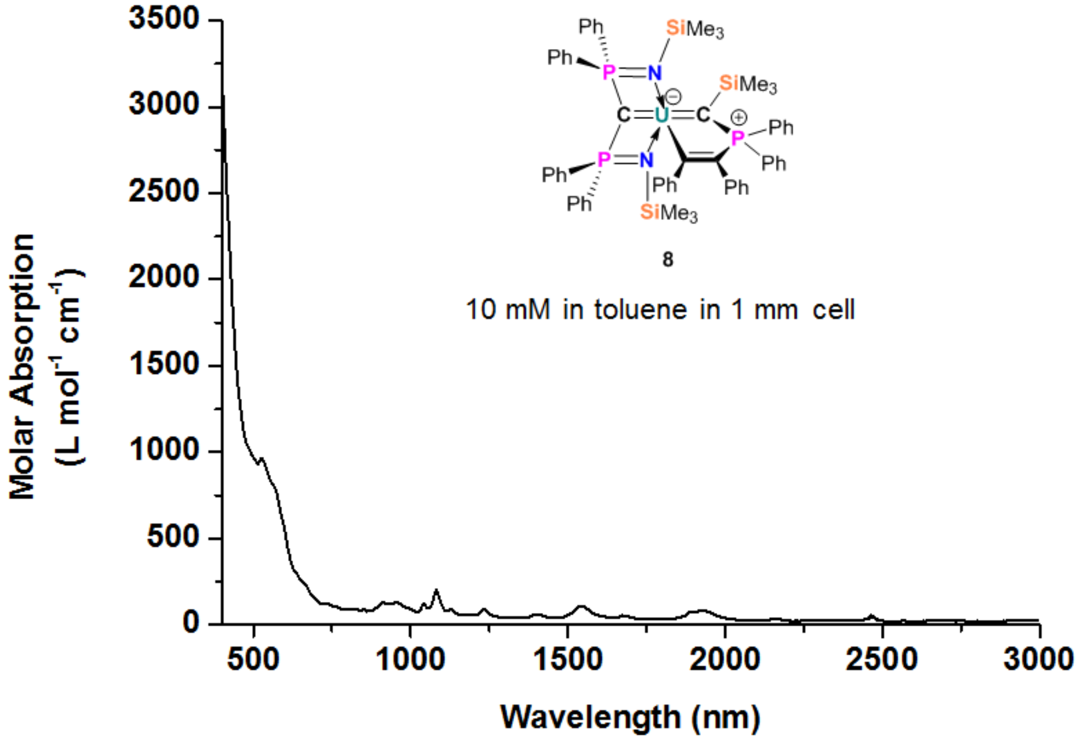

Figure S26. UV/Vis spectrum of **8**.

ATR-IR Spectra

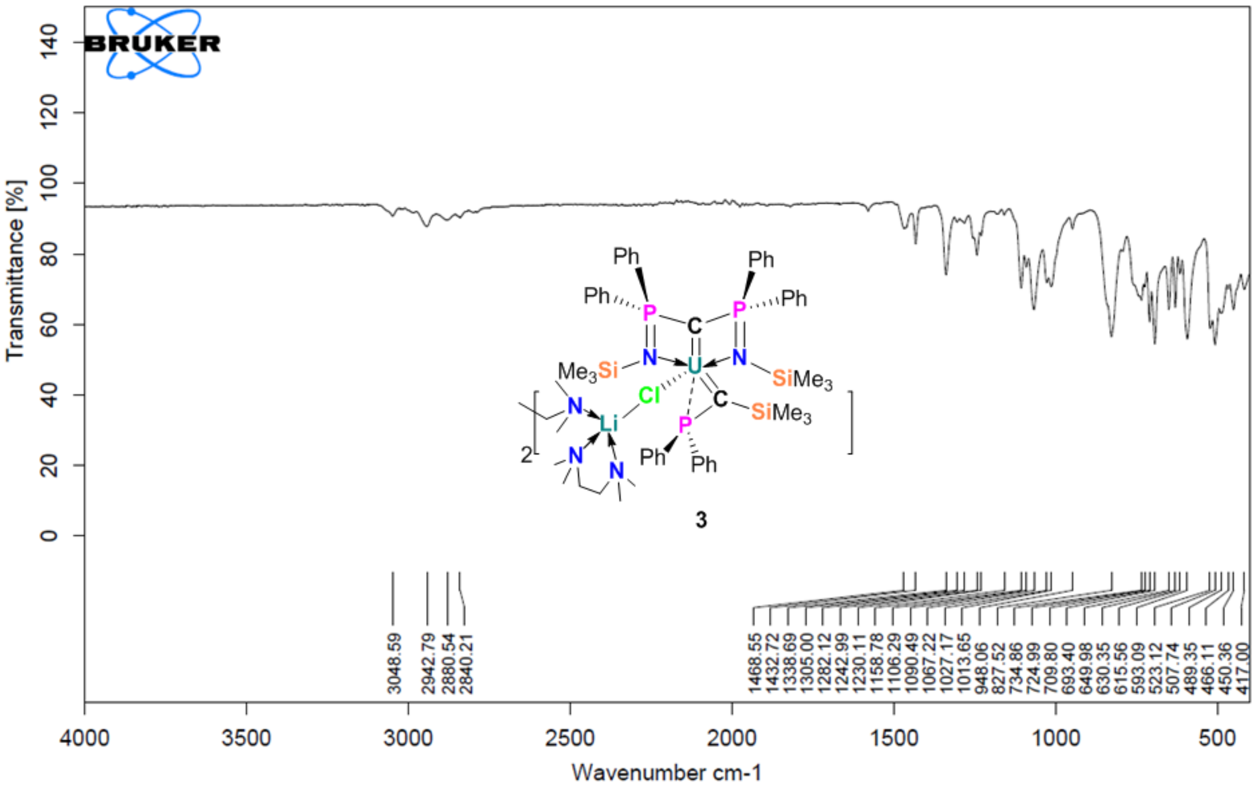

Figure S27. FTIR spectrum of **3**.

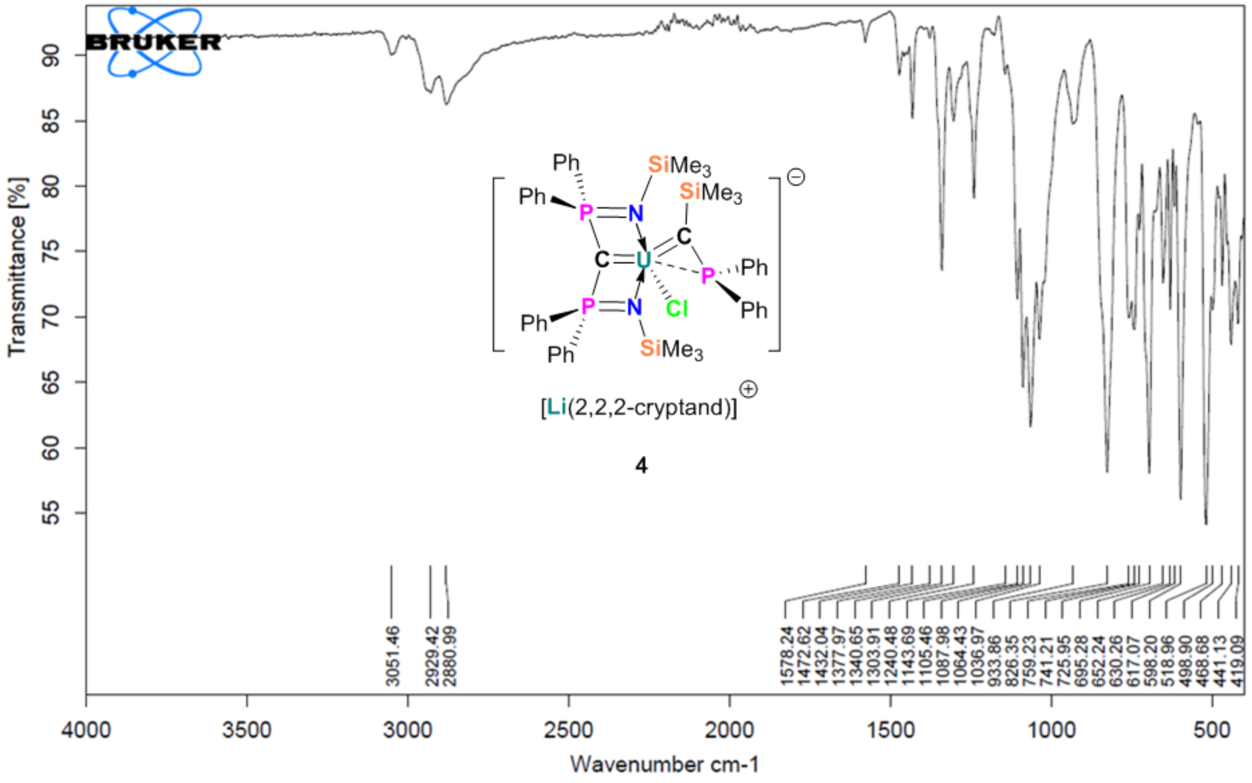

Figure S28. FTIR spectrum of **4**.

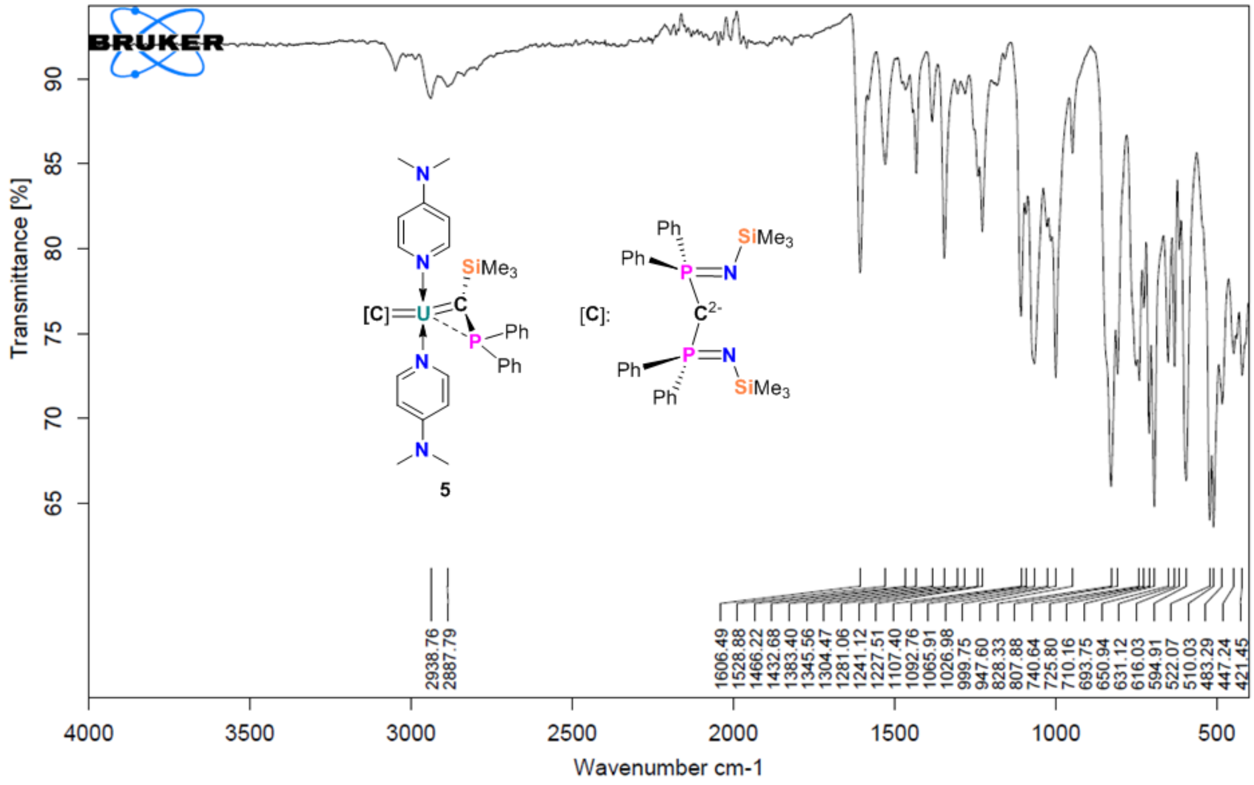

Figure S29. FTIR spectrum of **5**.

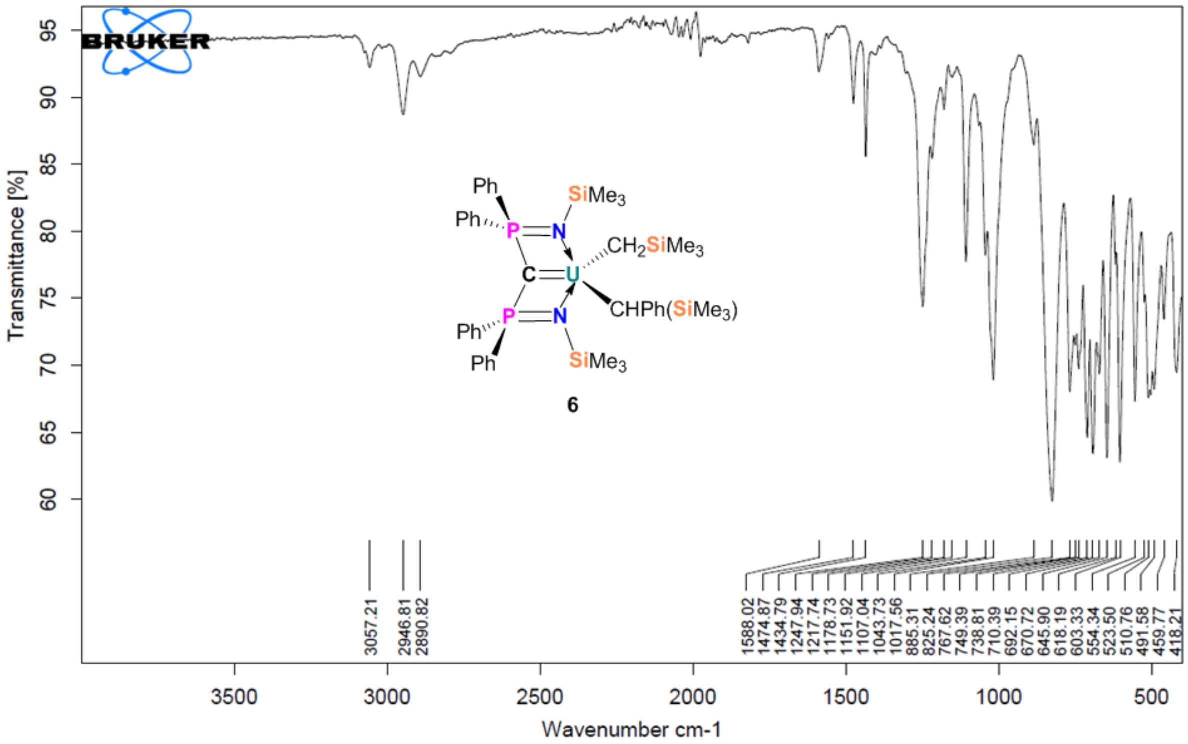

Figure S30. FTIR spectrum of **6**.

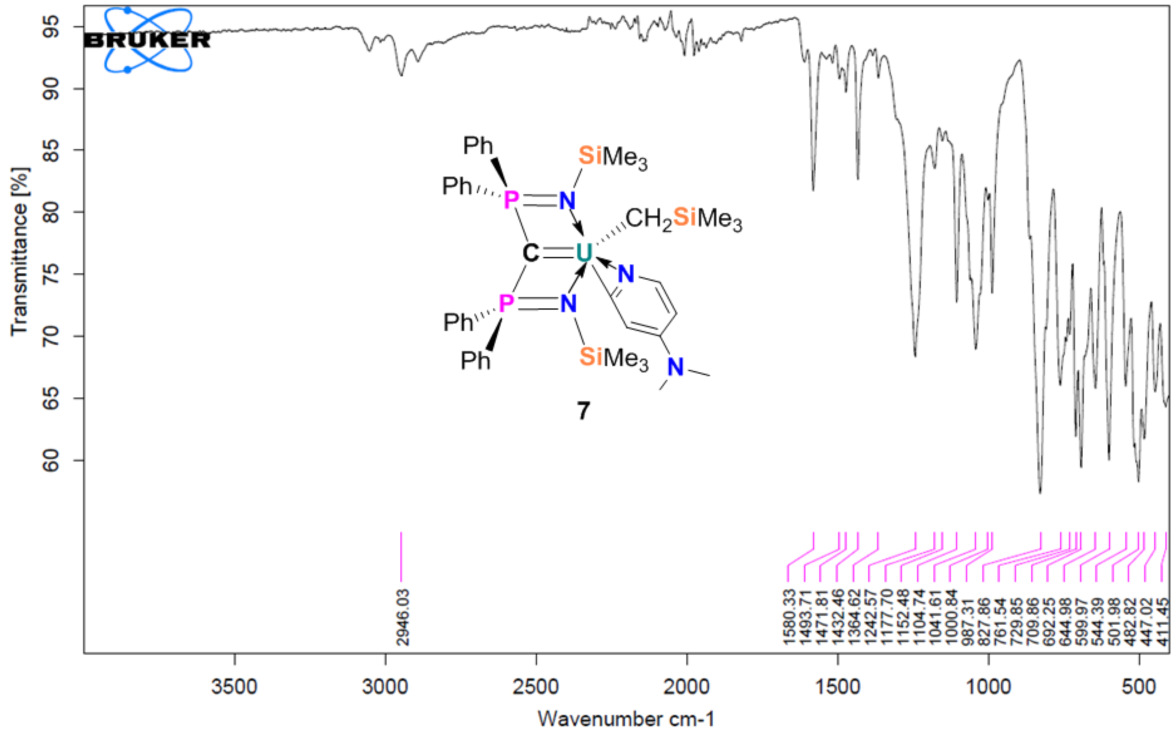

Figure S31. FTIR spectrum of **7**.

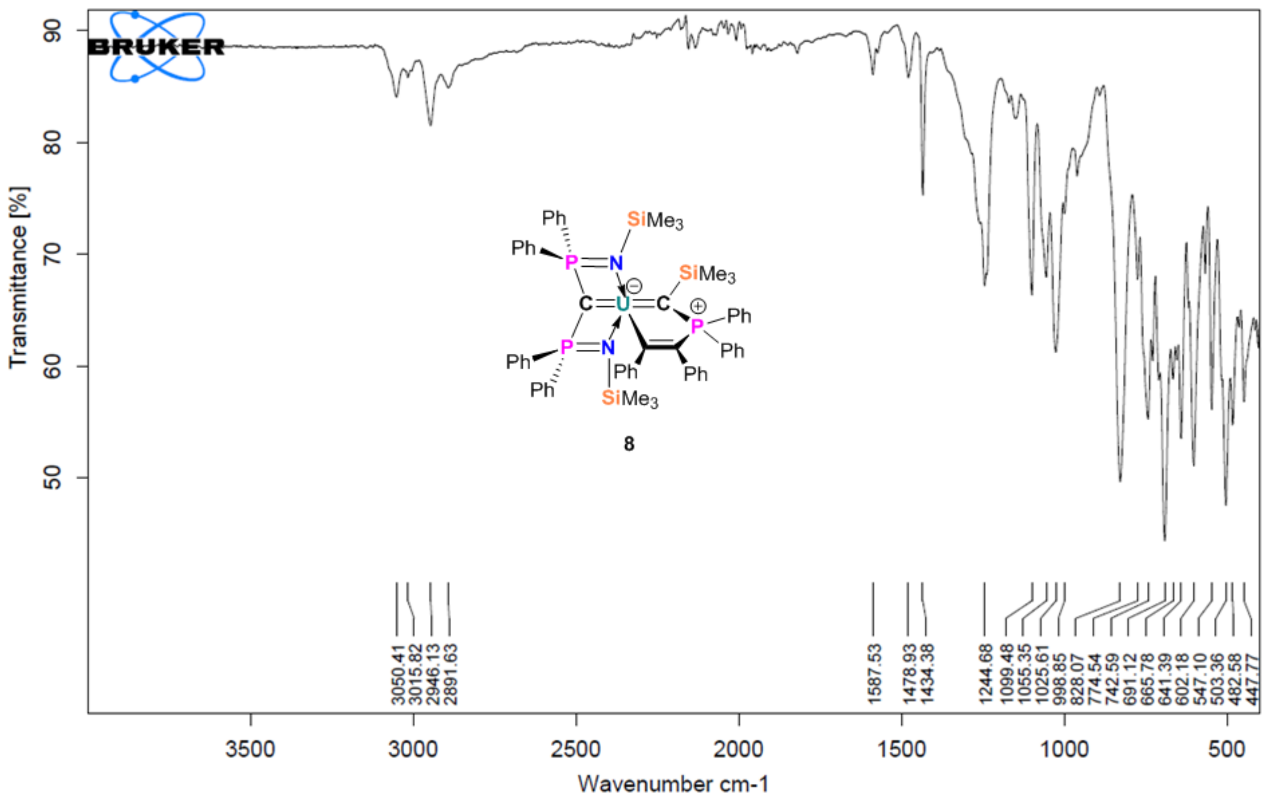

Figure S32. FTIR spectrum of **8**.

Magnetometric Measurements

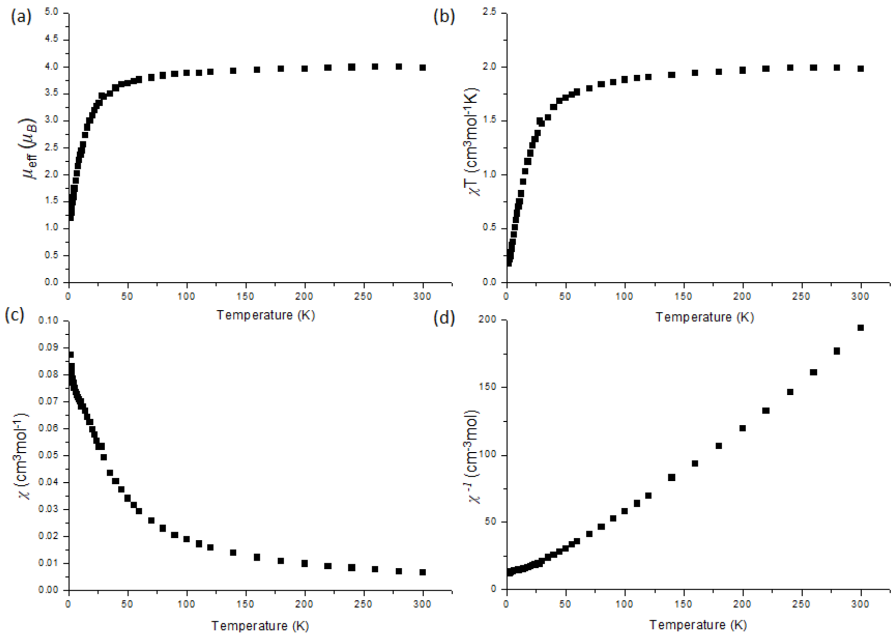

Figure S33. Magnetic data for powdered **3** in a 0.1 T applied magnetic field, presented as: (a) μ_{eff} vs T; (b) χT vs T; (c) χ vs T; (d) χ^{-1} vs T.

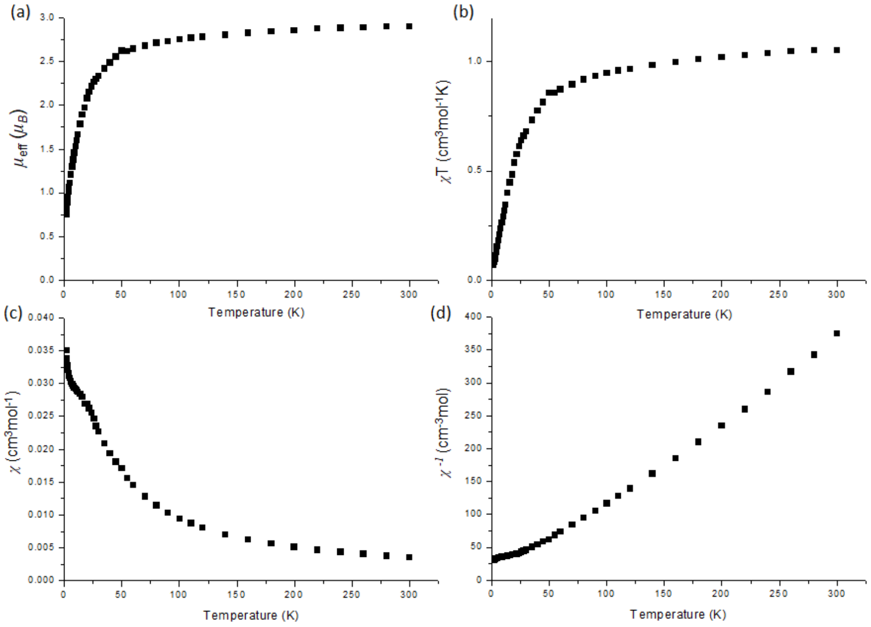

Figure S34. Magnetic data for powdered **4** in a 0.1 T applied magnetic field, presented as: (a) μ_{eff} vs T; (b) χT vs T; (c) χ vs T; (d) χ^{-1} vs T.

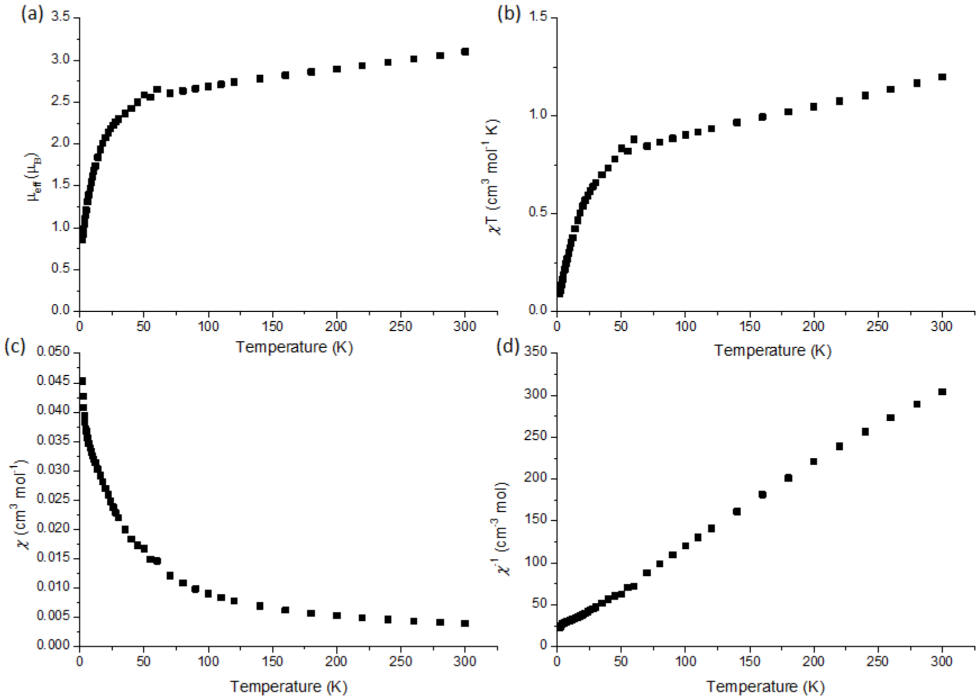

Figure S35. Magnetic data for powdered **5** in a 0.1 T applied magnetic field, presented as: (a) μ_{eff} vs T; (b) χT vs T; (c) χ vs T; (d) χ^{-1} vs T.

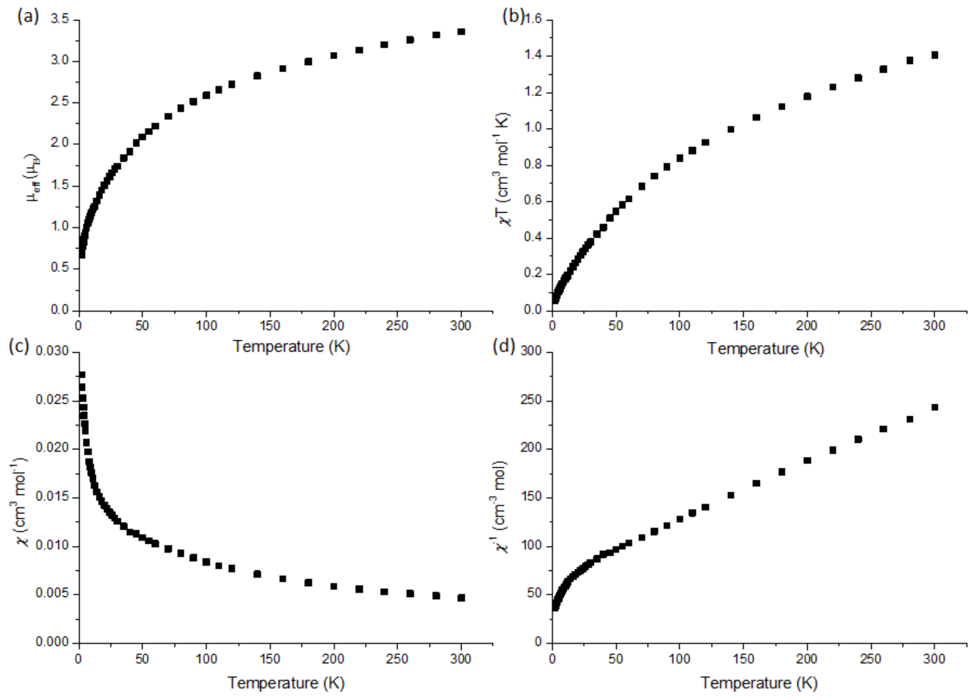

Figure S36. Magnetic data for powdered **6** in a 0.5 T applied magnetic field, presented as: (a) μ_{eff} vs T; (b) χT vs T; (c) χ vs T; (d) χ^{-1} vs T.

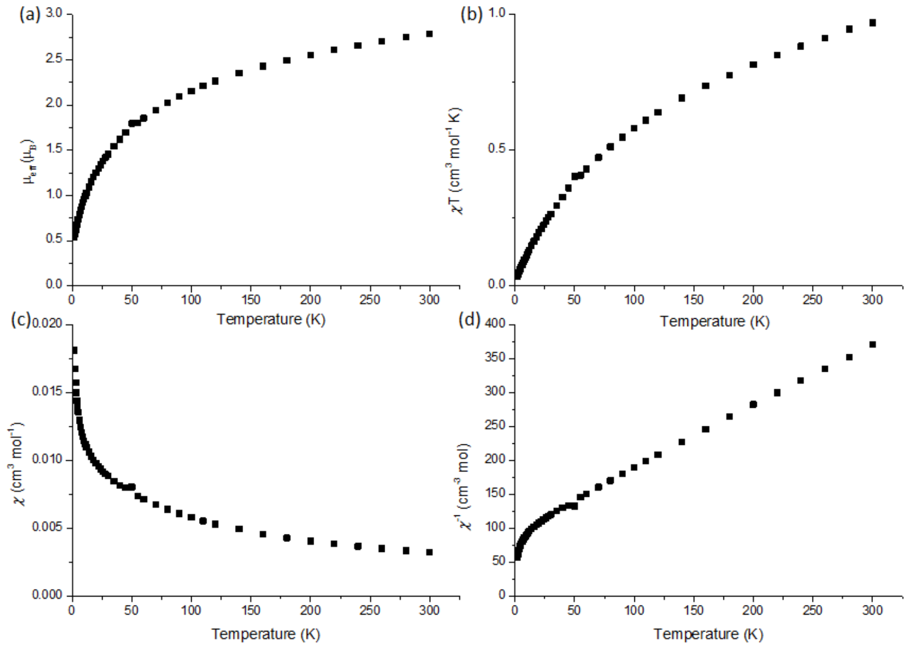

Figure S37. Magnetic data for powdered **7** in a 0.1 T applied magnetic field, presented as: (a) μ_{eff} vs T; (b) χT vs T; (c) χ vs T; (d) χ^{-1} vs T.

Computational Visualisations

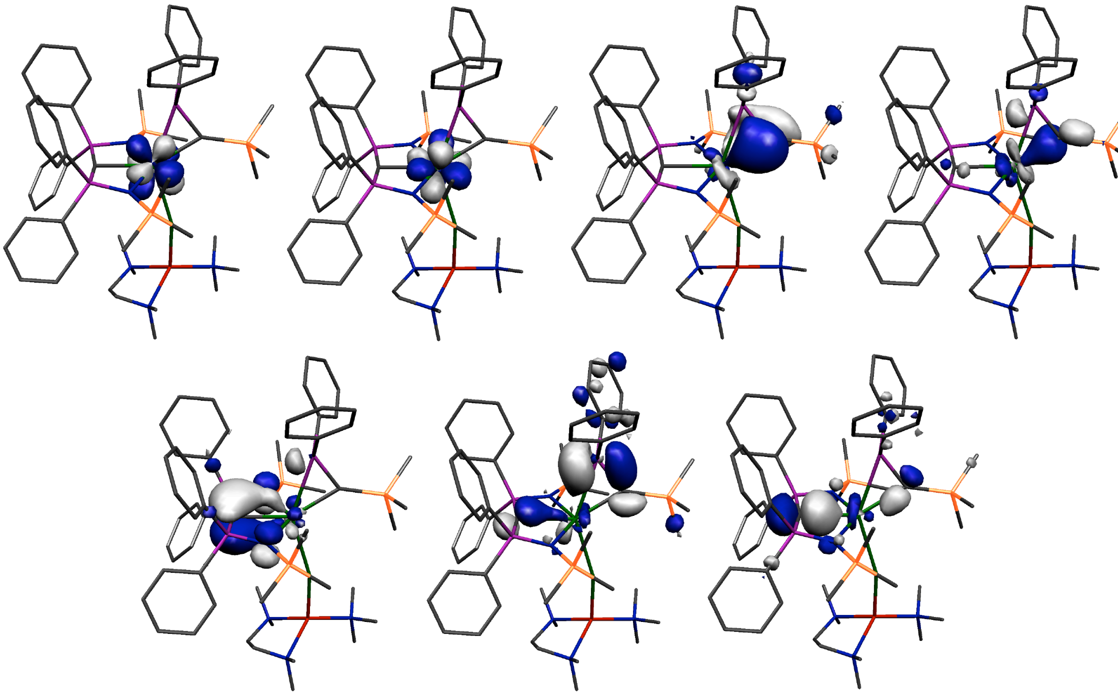

Figure S38. Top left to bottom right Kohn Sham Molecular Orbitals of **3'**: HOMO (−1.998 eV), HOMO−1 (−2.087 eV), HOMO−2 (−3.716 eV), HOMO−3 (−3.819 eV), HOMO−4 (−4.417 eV), HOMO−5 (−4.442 eV), and HOMO−6 (−5.124 eV). Hydrogen atoms are omitted for clarity.

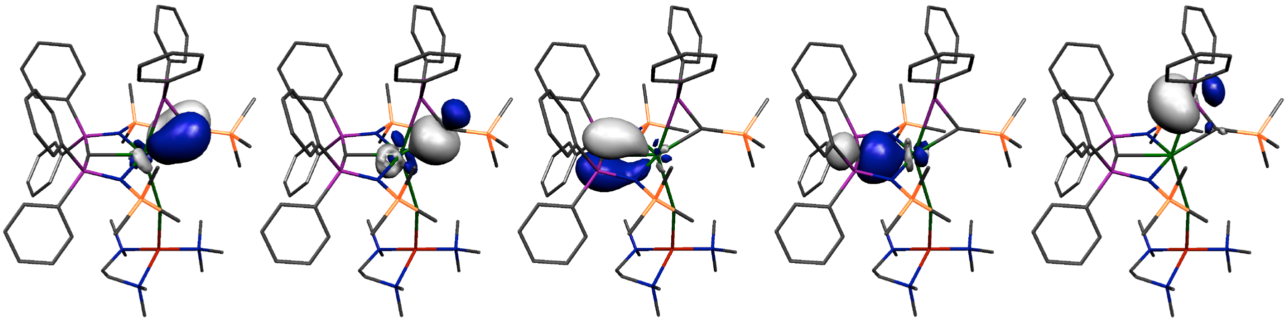

Figure S39. Left to right Natural Bond Orbitals of **3'**: U=$C_{carbene}$ π, U=$C_{carbene}$ σ, U=C_{BIPM} π, U=C_{BIPM} σ, and phosphine lone pair. Hydrogen atoms are omitted for clarity.

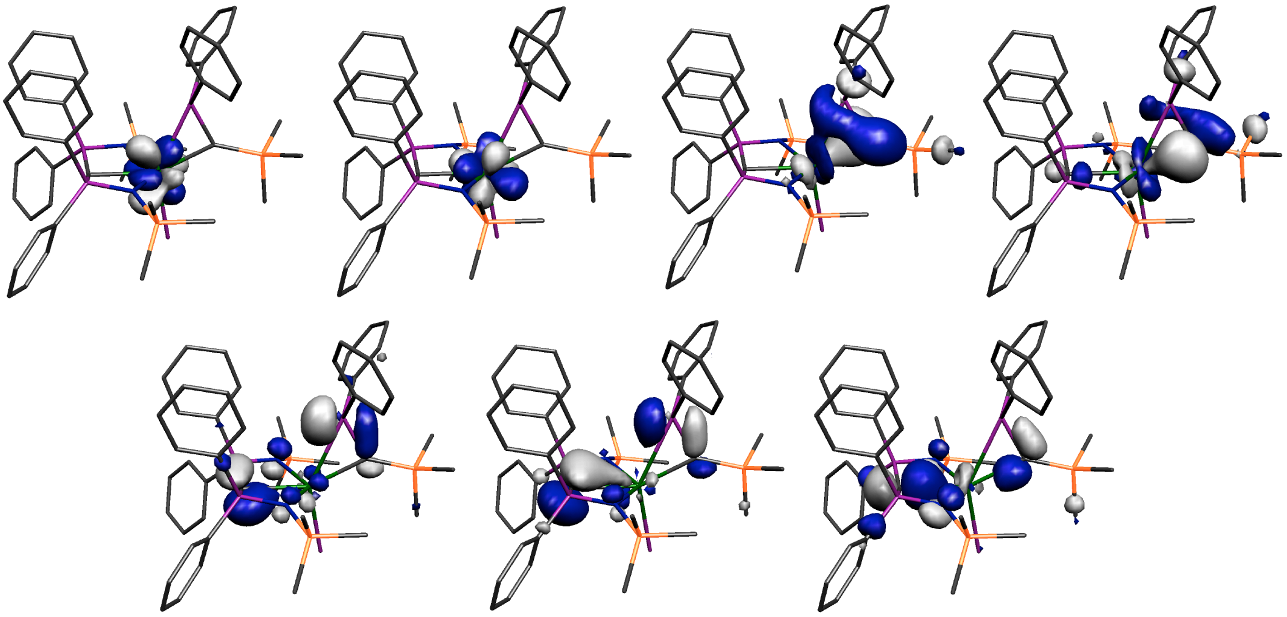

Figure S40. Top left to bottom right Kohn Sham Molecular Orbitals of **4**$^{-}$: HOMO (0.130 eV), HOMO−1 (0.004 eV), HOMO−2 (−1.636 eV), HOMO−3 (−1.815 eV), HOMO−4 (−2.338 eV), HOMO−5 (−2.494 eV), and HOMO−6 (−2.975 eV). Hydrogen atoms are omitted for clarity.

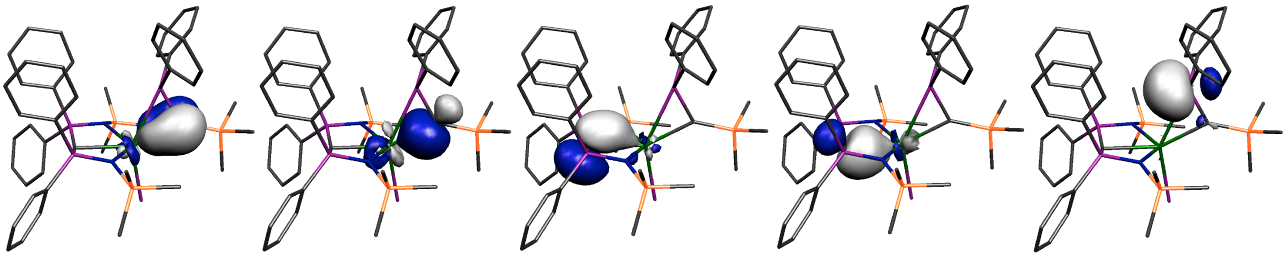

Figure S41. Left to right Natural Bond Orbitals of **4**$^{-}$: U=$C_{carbene}$ π, U=$C_{carbene}$ σ, U=C_{BIPM} π, U=C_{BIPM} σ, and phosphine lone pair. Hydrogen atoms are omitted for clarity.

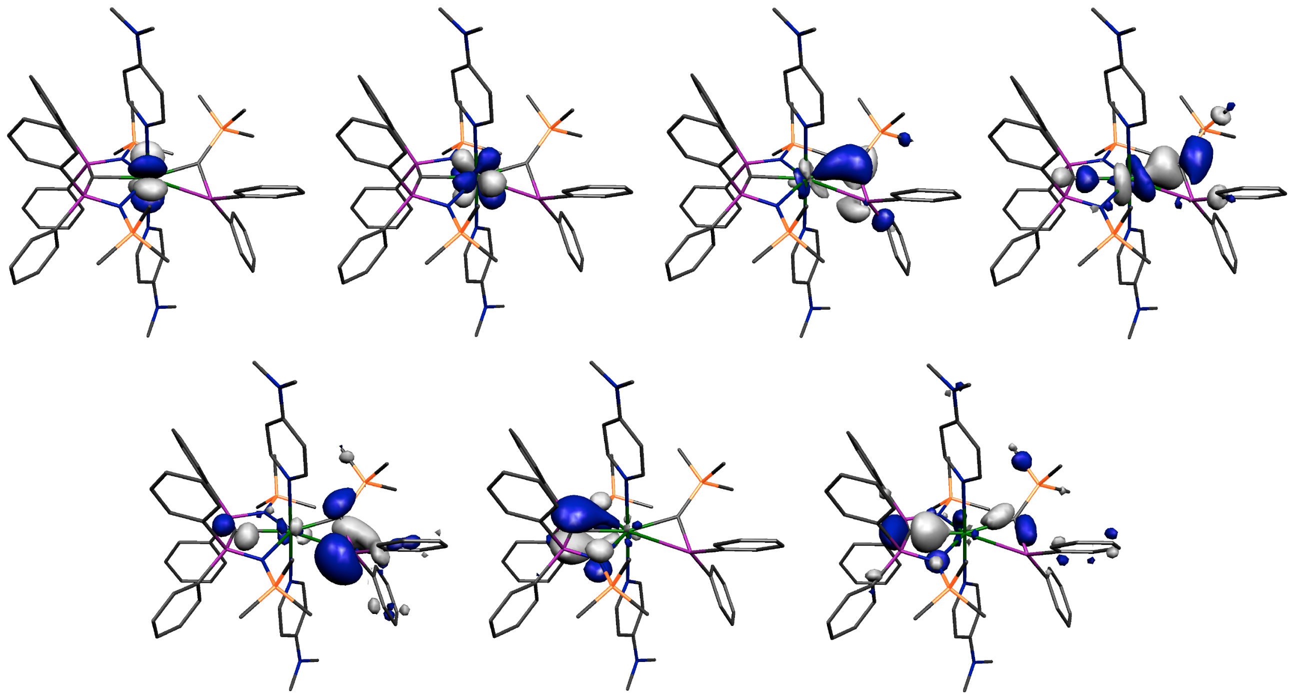

Figure S42. Top left to bottom right Kohn Sham Molecular Orbitals of **5**: HOMO (−1.731 eV), HOMO−1 (−2.278 eV), HOMO−2 (−3.815 eV), HOMO−3 (−4.196 eV), HOMO−4 (−4.389 eV), HOMO−5 (−4.453 eV), and HOMO−8 (−5.281 eV). HOMO−6 and HOMO−7 (not shown) are DMAP π-system contributions. Hydrogen atoms are omitted for clarity.

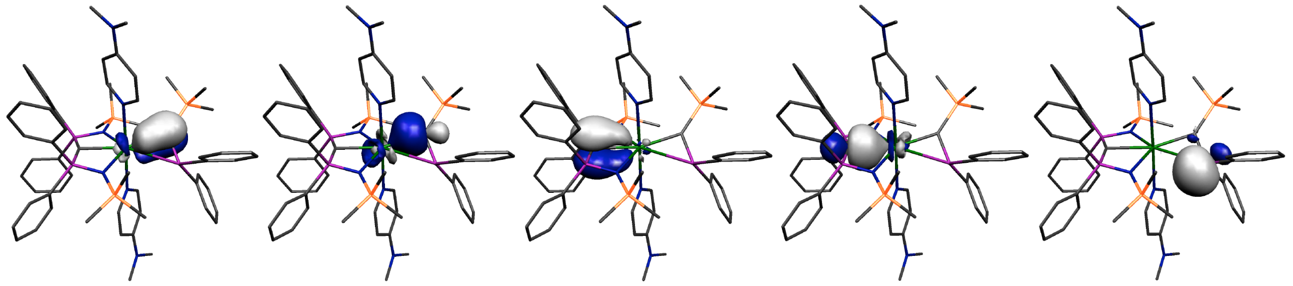

Figure S43. Left to right Natural Bond Orbitals of **5**: U=$C_{carbene}$ π, U=$C_{carbene}$ σ, U=C_{BIPM} π, U=C_{BIPM} σ, and phosphine lone pair. Hydrogen atoms are omitted for clarity.

Computed Data

Table S1. Final Coordinates and Single Point Energy of 3' After Geometry Optimisation

1.C	2.022473	1.848343	-5.211314
2.C	-4.628387	2.751231	-4.248407
3.C	-0.547619	-1.337222	-4.256836
4.C	-0.263575	3.480237	-4.032635
5.C	2.270948	-2.387916	-3.690118
6.C	2.605565	4.426616	-3.691811
7.C	6.052249	0.205867	-3.426865
8.C	-6.707721	3.183298	-3.097027
9.C	-0.139230	-4.187818	-3.277909
10.C	-6.990535	-0.930315	-2.814714
11.C	6.375327	-1.051751	-2.900200
12.C	-4.714371	-1.625286	-3.176121
13.C	4.993356	0.942214	-2.888907
14.C	-4.623165	3.979822	-2.173742
15.C	1.524686	1.890118	-2.120555
16.C	5.629024	-1.568241	-1.837540
17.C	4.230150	0.428240	-1.825109
18.C	4.560934	-0.834053	-1.309470
19.C	-5.520466	-1.388639	-0.912639
20.C	-2.185513	-3.548354	-0.506239
21.C	-3.225744	-4.456299	-0.288487
22.C	3.763169	2.717893	-0.230131
23.C	3.255944	4.025006	-0.176827
24.C	2.138228	-4.437533	-0.177230
25.C	-0.599014	4.211749	0.119298
26.C	-6.290382	-0.548886	0.104563
27.C	3.313711	-5.122367	0.138560
28.C	-1.034528	-3.572354	0.296129
29.C	4.967693	2.443139	0.447498
30.C	-6.964017	1.741925	0.534793
31.C	3.929877	5.032761	0.520214
32.C	-3.136915	-5.392771	0.748865
33.C	1.806543	-3.243831	0.487398
34.C	5.638029	3.445540	1.152527
35.C	5.123147	4.747321	1.192608
36.C	0.027658	-0.934221	0.991842
37.C	-4.716038	1.057362	1.048713
38.C	-0.955255	-4.512692	1.339365
39.C	4.168266	-4.627798	1.132800
40.C	-2.000231	-5.413118	1.565919
41.C	2.666554	-2.757048	1.481916
42.C	3.840440	-3.446590	1.804986
43.C	-2.109651	3.634596	2.675331
44.C	0.885300	4.089079	2.844950
45.C	2.520062	0.923314	3.047648
46.C	-2.391099	-1.102573	3.086609
47.C	1.366206	0.189634	3.367729
48.C	-1.483598	-0.068934	3.354187
49.C	3.659737	0.834684	3.851897

50.C	-3.466365	-1.343972	3.948900
51.C	-1.662050	0.716009	4.505901
52.C	1.378613	-0.645642	4.500943
53.C	3.662783	0.008926	4.981566
54.C	-3.647719	-0.546971	5.084507
55.C	2.520262	-0.733951	5.302422
56.C	-2.742047	0.485300	5.361623
57.H	1.945748	2.454174	-6.130088
58.H	3.058821	1.486593	-5.127590
59.H	1.380040	0.963263	-5.335269
60.H	-0.410708	-1.672021	-5.297411
61.H	-5.097449	1.930151	-4.807701
62.H	-4.722540	3.679728	-4.845636
63.H	2.317523	-2.773372	-4.722180
64.H	-0.308377	4.039770	-4.981094
65.H	2.591158	4.898088	-4.688906
66.H	-0.082354	-4.454170	-4.346938
67.H	6.632440	0.615730	-4.255664
68.H	-0.969731	2.638142	-4.099080
69.H	-7.005988	-0.567754	-3.850664
70.H	-3.563915	2.522770	-4.112672
71.H	-1.620727	-1.387071	-4.020380
72.H	-7.193444	2.371446	-3.655387
73.H	-0.247672	-0.279296	-4.208288
74.H	-4.765926	-1.222939	-4.195827
75.H	-6.871980	4.130226	-3.649710
76.H	2.689954	-1.370896	-3.686896
77.H	3.653888	4.199790	-3.443097
78.H	7.205147	-1.624209	-3.318615
79.H	2.922752	-3.015258	-3.063527
80.H	-7.387533	-1.966049	-2.804483
81.H	4.761386	1.927833	-3.294829
82.H	-0.615701	4.146119	-3.229750
83.H	-4.983715	-2.699746	-3.211958
84.H	-7.664700	-0.295848	-2.226086
85.H	-1.179690	-4.327609	-2.952353
86.H	2.255623	5.175306	-2.964725
87.H	0.477585	-4.920848	-2.736757
88.H	-4.701704	4.947658	-2.707040
89.H	-7.191222	3.268955	-2.114997
90.H	-3.682372	-1.520654	-2.820875
91.H	-3.562871	3.738027	-2.029238
92.H	5.869420	-2.547143	-1.419737
93.H	-5.098408	4.088259	-1.189202
94.H	-2.246978	-2.816965	-1.311780
95.H	-5.884907	-2.435251	-0.860946
96.H	-4.103048	-4.445855	-0.937520
97.H	-4.454055	-1.409569	-0.645824
98.H	1.472957	-4.839534	-0.940658
99.H	2.327497	4.246262	-0.701699
100.H	3.973134	-1.248846	-0.489591
101.H	0.201319	3.913722	-0.577092

102.H	-1.560394	3.983291	-0.368248
103.H	3.561806	-6.045173	-0.388306
104.H	-7.811069	1.629323	-0.155279
105.H	-6.637534	2.789961	0.516421
106.H	-0.545040	5.303686	0.256795
107.H	5.390780	1.437731	0.411294
108.H	3.522090	6.045011	0.536816
109.H	-3.944136	-6.108544	0.913705
110.H	-7.364345	-0.575430	-0.126884
111.H	-6.172904	-1.010178	1.103751
112.H	-3.873298	0.418078	0.762310
113.H	-4.371288	2.096788	0.990519
114.H	6.573406	3.211660	1.665148
115.H	5.650482	5.531393	1.738579
116.H	-7.315047	1.514663	1.561345
117.H	5.084952	-5.164878	1.383567
118.H	-0.068031	-4.545224	1.973967
119.H	-1.920398	-6.140226	2.375823
120.H	-4.998961	0.833510	2.094577
121.H	2.413489	-1.832354	2.001314
122.H	2.527062	1.557459	2.161105
123.H	1.866349	4.065797	2.348675
124.H	-2.944641	3.092446	2.209934
125.H	-2.332797	4.713229	2.616522
126.H	-2.233007	-1.724737	2.203547
127.H	4.496258	-3.054297	2.582969
128.H	0.635978	5.143061	3.052817
129.H	4.543640	1.417989	3.591156
130.H	0.988092	3.573807	3.812613
131.H	-2.088715	3.359765	3.739126
132.H	-4.153757	-2.165503	3.739907
133.H	-0.942757	1.498772	4.746775
134.H	0.494747	-1.231233	4.758354
135.H	4.551919	-0.054152	5.611892
136.H	-4.483979	-0.736506	5.760146
137.H	2.515349	-1.382398	6.179835
138.H	-2.870937	1.101846	6.252538
139.Cl	-2.484693	0.955707	-1.773133
140.Li	-4.861489	1.113542	-1.824468
141.N	-5.274685	2.886660	-2.925495
142.N	-5.614247	-0.858108	-2.294646
143.N	0.297135	-1.727998	-1.473915
144.N	-5.852429	0.869950	0.119366
145.N	-0.159306	1.629577	1.394505
146.P	2.811175	1.362144	-1.073577
147.P	0.283525	-2.302807	0.062990
148.P	-0.064734	0.205694	2.213526
149.Si	1.507678	2.865460	-3.683775
150.Si	0.490922	-2.404399	-3.087018
151.Si	-0.466056	3.312052	1.781025
152.U	0.150027	0.630410	-0.813634

Energy: −880.42216841 eV

Table S2. Final Coordinates and Single Point Energy of 4^- After Geometry Optimisation

1.C	-4.181266	-2.052894	-5.141246
2.C	0.225889	-2.001052	-5.046791
3.C	-3.855725	-0.729625	-4.817488
4.C	-3.690247	-3.100548	-4.353302
5.C	-3.037891	-0.458100	-3.717161
6.C	2.758042	-1.779820	-3.429050
7.C	-2.873832	-2.826172	-3.252828
8.C	0.858068	-4.132154	-2.891351
9.C	-2.534925	-1.501177	-2.927960
10.C	4.744864	2.140075	-2.524116
11.C	-4.248855	4.045923	-1.965911
12.C	-3.288166	3.097618	-1.607072
13.C	6.035367	-0.348132	-1.374580
14.C	-4.958560	4.735527	-0.975124
15.C	-1.446365	0.481254	-1.051847
16.C	-0.662391	5.350256	-0.639655
17.C	3.120463	0.507539	-0.487009
18.C	-3.018963	2.828023	-0.256520
19.C	-1.803841	-2.391827	-0.308570
20.C	2.049485	4.299495	0.114937
21.C	-4.694508	4.476957	0.374257
22.C	-3.149752	-2.617336	0.037453
23.C	5.682761	2.122944	0.371418
24.C	3.780906	-2.688993	0.384458
25.C	4.070510	-4.044085	0.583867
26.C	-0.797645	-3.095621	0.368797
27.C	-3.726399	3.532477	0.731919
28.C	-3.478846	-3.534472	1.038671
29.C	2.952364	-1.986649	1.272204
30.C	-1.128803	-4.014903	1.369806
31.C	3.526870	-4.726586	1.677647
32.C	-2.468103	-4.236506	1.707642
33.C	-2.161768	0.973053	1.783101
34.C	-3.483530	0.557127	2.030614
35.C	-0.127010	4.622973	2.294339
36.C	2.395349	-2.689675	2.358799
37.C	2.683713	-4.041666	2.563979
38.C	3.053960	0.568700	2.531448
39.C	2.513191	1.822230	2.864124
40.C	-1.181421	0.734067	2.758474
41.C	-3.814720	-0.075893	3.231767
42.C	4.044783	0.032650	3.375759
43.C	2.951534	2.531078	3.984648
44.C	-1.512332	0.096114	3.958270
45.C	-2.829101	-0.311321	4.198147
46.C	4.485717	0.737282	4.501470
47.C	3.945863	1.990678	4.810595
48.H	-4.814510	-2.267121	-6.004489
49.H	0.834622	-2.550690	-5.784997

50.H	-4.237215	0.092285	-5.426529
51.H	0.271088	-0.930913	-5.302899
52.H	-0.817430	-2.332774	-5.154162
53.H	-3.939367	-4.134932	-4.597969
54.H	3.273110	-2.412347	-4.171370
55.H	1.344152	-4.730776	-3.679893
56.H	2.857989	-0.734109	-3.761362
57.H	-2.772855	0.565918	-3.453588
58.H	4.293815	1.605880	-3.373438
59.H	-4.440813	4.250741	-3.020898
60.H	5.761295	2.452387	-2.816467
61.H	-0.178778	-4.488814	-2.793684
62.H	-2.497523	-3.646688	-2.640829
63.H	3.277965	-1.872194	-2.466903
64.H	4.144004	3.047737	-2.363496
65.H	-2.720686	2.560823	-2.367662
66.H	5.641718	-0.992634	-2.176306
67.H	1.372214	-4.339990	-1.940543
68.H	6.994609	0.070186	-1.724225
69.H	-5.712405	5.474304	-1.254467
70.H	-0.526951	5.064854	-1.694352
71.H	2.225610	4.167582	-0.964897
72.H	-0.199904	6.341988	-0.497353
73.H	6.246100	-0.981576	-0.498941
74.H	-1.740769	5.448765	-0.443353
75.H	4.202630	-2.154130	-0.465979
76.H	-3.942662	-2.074383	-0.480067
77.H	4.727323	-4.565288	-0.116058
78.H	6.683563	2.433825	0.026438
79.H	2.376491	5.318831	0.380242
80.H	0.246381	-2.916050	0.112135
81.H	2.692179	3.585046	0.646969
82.H	5.102506	3.032869	0.590240
83.H	-5.242041	5.010341	1.152521
84.H	5.800680	1.573176	1.318534
85.H	-4.526221	-3.699348	1.300309
86.H	-4.256627	0.725008	1.278901
87.H	-3.526276	3.337489	1.786060
88.H	3.754288	-5.783012	1.838080
89.H	-0.331608	-4.551587	1.886358
90.H	0.237786	5.648222	2.472438
91.H	1.742393	2.243760	2.220170
92.H	-2.724841	-4.951466	2.492734
93.H	-1.205066	4.609675	2.519242
94.H	-0.151740	1.031059	2.564045
95.H	0.367679	3.957422	3.016622
96.H	1.728311	-2.171167	3.049521
97.H	2.247907	-4.565035	3.418044
98.H	4.481426	-0.940982	3.148064
99.H	-4.842770	-0.398416	3.407918
100.H	2.518049	3.506609	4.212906
101.H	-0.733999	-0.090867	4.700211

102.H	5.263719	0.305398	5.134918
103.H	-3.084908	-0.821384	5.129736
104.H	4.296452	2.544031	5.684568
105.Cl	1.059241	2.296773	-3.421705
106.N	0.192637	-1.302069	-2.068125
107.N	-0.222108	2.427510	0.219962
108.P	-1.362276	-1.118428	-1.558275
109.P	-1.667153	1.640870	0.142062
110.P	2.471385	-0.221004	0.950576
111.Si	0.941118	-2.284833	-3.315387
112.Si	4.812622	1.065991	-0.954220
113.Si	0.207217	4.108165	0.494173
114.U	0.972864	0.824456	-1.202800

Energy: −684.90946927 eV

Table S3. Final Coordinates and Single Point Energy of 5 After Geometry Optimisation

1.C	2.596926	-1.326768	-7.045991
2.C	1.208333	-3.365537	-6.538780
3.C	-4.072163	1.392106	-4.927462
4.C	-2.675483	1.378148	-4.839165
5.C	1.341627	-1.504886	-4.951346
6.C	1.785945	-0.219203	-4.545714
7.C	5.895825	-0.750588	-3.851200
8.C	0.578418	-2.227479	-3.997296
9.C	-4.838263	1.538839	-3.765394
10.C	5.581797	-1.950658	-3.196310
11.C	-2.048625	1.521018	-3.598025
12.C	5.461900	0.462889	-3.306295
13.C	1.527208	0.222795	-3.261299
14.C	-3.877235	-2.757533	-2.972682
15.C	-0.626003	4.632160	-2.923674
16.C	0.374022	-1.696122	-2.736542
17.C	-4.773097	-3.726798	-2.507187
18.C	-4.211284	1.680251	-2.524632
19.C	-2.807557	1.682254	-2.428701
20.C	4.844993	-1.932131	-2.010677
21.C	4.719627	0.475965	-2.120076
22.C	-3.103622	-2.023076	-2.069382
23.C	2.018812	3.714047	-1.787146
24.C	4.409114	-0.716197	-1.448806
25.C	-4.891820	-3.955500	-1.132207
26.C	-3.507404	4.123069	-0.391038
27.C	-3.205197	-2.253521	-0.687107
28.C	-4.113541	-3.225274	-0.230163
29.C	1.329607	-4.091921	-0.057359
30.C	-4.323785	4.980673	0.349902
31.C	-0.033316	5.184382	0.005973
32.C	-3.077805	2.897178	0.148085
33.C	-1.767842	0.262074	-0.050547
34.C	-1.455270	-4.974080	0.705987
35.C	5.328184	2.708556	0.607614

36.C	2.766363	0.853809	0.557564
37.C	5.795692	-1.497655	1.241832
38.C	4.391991	-1.587451	1.263219
39.C	-4.729028	4.622337	1.642242
40.C	-3.499877	2.542179	1.437513
41.C	6.568891	-2.115073	2.228681
42.C	-4.317752	3.399901	2.182121
43.C	2.795277	3.671500	1.920274
44.C	3.794048	-2.330785	2.294122
45.C	-4.459723	-0.915144	2.019717
46.C	-3.105485	-1.299873	2.020068
47.C	0.317766	-3.850210	2.847495
48.C	5.956153	-2.840254	3.257929
49.C	4.561224	-2.948399	3.285414
50.C	-0.474970	1.892968	2.960186
51.C	4.352468	1.456796	3.225015
52.C	-5.181560	-0.856126	3.213993
53.C	-2.491923	-1.607763	3.241285
54.C	0.887377	0.132837	3.529007
55.C	-0.749033	2.179046	4.285681
56.C	-4.560869	-1.177246	4.428874
57.C	-3.213566	-1.550195	4.438766
58.C	0.667442	0.326423	4.880740
59.C	-0.184552	1.378558	5.311254
60.C	-1.459309	2.588744	7.015392
61.C	0.025473	0.669129	7.647062
62.H	2.855502	-1.973630	-7.890335
63.H	1.571663	-3.608754	-7.541908
64.H	2.177554	-0.390646	-7.449833
65.H	0.109407	-3.438950	-6.549283
66.H	3.522778	-1.086025	-6.499265
67.H	1.598266	-4.122235	-5.836917
68.H	-4.562313	1.291099	-5.897484
69.H	-2.070203	1.262176	-5.739476
70.H	6.478912	-0.764422	-4.774189
71.H	2.363397	0.420437	-5.210051
72.H	0.166328	-3.209621	-4.219452
73.H	5.912885	-2.904060	-3.612818
74.H	-5.927970	1.546018	-3.823387
75.H	5.707908	1.404833	-3.801527
76.H	-3.790857	-2.557811	-4.041700
77.H	-0.462038	4.006216	-3.813065
78.H	-0.961684	1.508673	-3.530126
79.H	-5.383384	-4.294470	-3.212151
80.H	-0.164984	5.615486	-3.117489
81.H	1.893974	1.194644	-2.936784
82.H	-1.709687	4.794671	-2.833834
83.H	2.152805	3.380541	-2.827580
84.H	-2.428939	-1.249610	-2.436854
85.H	-0.183168	-2.257836	-1.988794
86.H	4.604149	-2.872745	-1.511519
87.H	-4.819707	1.794838	-1.626276

88.H	4.375949	1.418946	-1.694281
89.H	2.536757	4.680372	-1.688023
90.H	-3.214394	4.406378	-1.401497
91.H	1.024299	-4.246820	-1.103882
92.H	-5.592474	-4.703150	-0.757058
93.H	2.515554	2.987709	-1.126096
94.H	-1.827300	-4.980356	-0.329287
95.H	-4.647834	5.928102	-0.083283
96.H	5.063424	3.259825	-0.308307
97.H	0.463959	6.126879	-0.276861
98.H	2.104419	-3.310759	-0.052021
99.H	-1.096478	5.407408	0.184353
100.H	1.779115	-5.031797	0.300561
101.H	6.003071	1.888096	0.321622
102.H	-0.974549	-5.949273	0.894575
103.H	6.286734	-0.936455	0.445503
104.H	-4.218162	-3.414623	0.838125
105.H	-2.323359	-4.900526	1.377594
106.H	0.404072	4.849088	0.956779
107.H	5.892591	3.399287	1.256499
108.H	2.536177	4.241163	1.018156
109.H	-4.953039	-0.664238	1.079208
110.H	-5.369988	5.290887	2.220199
111.H	-3.189846	1.581130	1.849171
112.H	7.656131	-2.027565	2.193775
113.H	2.709892	-2.443769	2.303324
114.H	-0.915556	2.502533	2.173183
115.H	1.866516	3.457216	2.470263
116.H	3.411087	4.326502	2.558326
117.H	0.920698	-4.764192	2.968987
118.H	-4.640638	3.103557	3.181394
119.H	4.951860	0.536591	3.158167
120.H	0.898078	-3.012191	3.259571
121.H	-0.582748	-3.968251	3.469244
122.H	-6.232654	-0.563492	3.197311
123.H	-1.440705	-1.889105	3.246445
124.H	6.561306	-3.325506	4.025523
125.H	1.567704	-0.649405	3.194989
126.H	4.072162	-3.528899	4.069934
127.H	4.968142	2.230899	3.713318
128.H	3.493077	1.255146	3.883116
129.H	-1.408697	3.014712	4.507680
130.H	-5.126946	-1.137246	5.361580
131.H	-2.720348	-1.800434	5.379678
132.H	1.166436	-0.332183	5.588341
133.H	-1.216461	3.584099	6.617359
134.H	-2.461171	2.303419	6.650057
135.H	1.116866	0.550048	7.598336
136.H	-0.438497	-0.326526	7.532957
137.H	-1.496131	2.664978	8.106576
138.H	-0.223805	1.054114	8.640904
139.N	1.644817	-2.021100	-6.188075

140.N	0.846552	-0.490698	-2.331278
141.N	-0.426941	2.313217	-0.906715
142.N	-0.641840	-1.977701	0.667902
143.N	0.320292	0.876307	2.551136
144.N	-0.439388	1.615820	6.642221
145.P	-1.972299	1.751627	-0.788874
146.P	3.296262	-0.730372	0.032757
147.P	-2.118257	-1.299196	0.460913
148.Si	0.179830	3.899764	-1.367315
149.Si	-0.155867	-3.629266	1.013021
150.Si	3.772381	2.088143	1.524804
151.U	0.621413	0.263163	0.091328

Energy: −907.14570809 eV

References

1. O. J. Cooper, D. P. Mills, J. McMaster, F. Moro, E. S. Davies, W. Lewis, A. J. Blake, S. T. Liddle, *Angew. Chem. Int. Ed.* **2011**, *50*, 2383.
2. D. J. Peterson, *J. Org. Chem.* **1968**, *33*, 780.
3. W. Zarges, M. Marsch, K. Harms, G. Frenking, G. Boche, *Chem. Ber.* **1991**, *124*, 543.
4. D. P. Mills, O. J. Cooper, F. Tuna, E. J. L. McInnes, E. S. Davies, J. McMaster, F. Moro, W. Lewis, A. J. Blake, S. T. Liddle, *J. Am. Chem. Soc.* **2012**, *134*, 10047.
5. H. -F. Hu, C. -M. Cui, *Organometallics* **2012**, *31*, 1208.
6. P. Coppens, W. C. Hamilton, *Acta Cryst. Sect. A* **1970**, *26*, 71.
7. G. M. Sheldrick, *Acta Cryst. Sect. A* **2015**, *71*, 3.
8. L. Palatinus, G. Chapuis, *J. Appl. Cryst.* **2007**, *40*, 786.
9. CrysAlisPRO, Oxford Diffraction /Agilent Technologies UK Ltd, Yarnton, England 2010.
10. O. V. Dolomanov, L. J. Bourhis, R. J. Gildea, J. A. K. Howard, H. Puschmann, *J. Appl. Cryst.* **2009**, *42*, 339.
11. L. J. Farugia, *J. Appl. Cryst*. **2012**, *45*, 849.
12. Persistence of Vision (TM) Raytracer, Persistence of Vision Pty. Ltd., Williamstown, Victoria, Australia, 2004.
13. C. Fonseca Guerra, J. G. Snijders, G. Te Velde, E. J. Baerends, *Theor. Chem. Acc.* **1998**, *99*, 391.

14. G. Te Velde, F. M. Bickelhaupt, S. J. van Gisbergen, A. C. Fonseca Guerra, E. J. Baerends, J. G. Snijders, T. Ziegler, *J. Comput. Chem.* **2001**, *22*, 931.
15. S. H. Vosko, L. Wilk, M. Nusair, *Can. J. Phys.* **1980**, *58*, 1200.
16. A. D. Becke, *Phys. Rev. A.* **1988**, *38*, 3098.
17. J. P. Perdew, *Phys. Rev. B.* **1986**, *33*, 8822.
18. S. Portmann, H. P. Luthi, *Chimia* **2000**, *54*, 766.
19. NBO 5.0: E. D. Glendening, J. K. Badenhoop, A. E. Reed, J. E. Carpenter, J. A. Bohmann, C. M. Morales, F. Weinhold, (Theoretical Chemistry Institute, University of Wisconsin, Madison, WI, 2001); http://www.chem.wisc.edu/~nbo5.
20. R. F. W. Bader, *Atoms in Molecules: A Quantum Theory*, Oxford University Press, New York, 1990.
21. R. F. W. Bader, *J. Phys. Chem. A* **1998**, *102*, 7314.
22. http://www.quimica.urv.es/XAIM.
